# Molecular analysis of axonal-intrinsic and glial-associated co-regulation of axon degeneration

**DOI:** 10.1038/cddis.2017.489

**Published:** 2017-11-09

**Authors:** Alejandra Catenaccio, Maica Llavero Hurtado, Paula Diaz, Douglas J Lamont, Thomas M Wishart, Felipe A Court

**Affiliations:** 1 Center for Integrative Biology, Faculty of Sciences, Universidad Mayor, Santiago 8580745, Chile; 2 FONDAP Center for Geroscience, Brain Health and Metabolism, Santiago, Chile; 3 The Roslin Institute, University of Edinburgh, Easter Bush, Midlothian EH25 9RG, UK; 4 FingerPrints Proteomics Facility, School of Life Sciences, University of Dundee, Dundee DD1 5EH, UK; 5 Euan MacDonald Centre for Motor Neuron Disease Research, University of Edinburgh, Edinburgh EH25 9RG, UK

## Abstract

Wallerian degeneration is an active program tightly associated with axonal degeneration, required for axonal regeneration and functional recovery after nerve damage. Here we provide a functional molecular foundation for our undertstanding of the complex non-cell autonomous role of glial cells in the regulation of axonal degeneration. To shed light on the complexity of the molecular machinery governing axonal degeneration we employ a multi-model, unbiased, *in vivo* approach combining morphological assesment and quantitative proteomics with *in silico-*based higher order functional clustering to genetically uncouple the intrinsic and extrinsic processes governing Wallerian degeneration. Highlighting a pivotal role for glial cells in the early stages fragmenting the axon by a cytokinesis-like process and a cell autonomous stage of axonal disintegration associated to mitochondrial dysfunction.

Axonal degeneration is a common feature of neurodegenerative conditions,^1^ representing a target for protective interventions. A variety of stimuli can trigger axonal degeneration, including genetic, toxic and mechanical insults.^2^ Once axons are disconnected from their cell bodies, they degenerate in an orderly fashion. After a delay of 1 to 2 days, they rapidly disintegrate over 24 hours. Importantly, axonal degeneration is required for regeneration in the peripheral nervous system.^3^

To date, much of our knowledge regarding the mechanism of axonal degeneration and its role in neurodegenerative conditions has been elucidated through the study of models with altered neuronal vulnerability. The foremost of these is the spontaneous neuroprotective mutation Wld^s^. In *Drosophila* and rodents, expressing the product of this mutation is sufficient to delay axonal degeneration by weeks.^4^ This suggests a high degree of evolutionary conservation of the molecular cascades governing axonal degeneration. Given this conservation in post-initiation degenerative cascades, injury-induced degeneration becomes an attractive system in which to study the processes underpinning axonal degeneration.^2^

Such experimental systems can be simplified by examining the process in isolated neurons in culture *in vitro* to remove ‘confounding’ factors such as myelinating cells and immune response mediators. In such preparations, axons degenerate in a manner similar to axons undergoing Wallerian degeneration *in vivo*.^5^ This apparent similarity between *in vitro* and *in vivo* ‘degeneration’ has led to the notion that degeneration would be entirely mediated by the neuronal axon.^6^ Therefore, the participation of other cellular types in this degenerative process, especially glial cells, has been largely overlooked. Given that axonal degeneration occurs in a range of conditions where the initiating insult appears to be extrinsic to the axon (i.e., immune mediated/demyelinating neuropathies) such *in vitro* approaches are not sufficient to identify regulators, which would be effective *in vivo*. In addition, the process of degeneration seems to be under regulation of several intra-axonal organelles as axonal degeneration is associated with calcium release from the axonal endoplasmic reticulum^7^ and activation of the permeability transition pore (mPTP^5^), suggesting an orchestration between non-cell autonomous as well as cell autonomous events.

Glial cells participate in processes which are crucial for the function of their associated neurons, including but not limited to energetic metabolism^8, 9^ and the segregation of distinct axonal domains.^10^ Glial cells are also proven to contribute to non-autonomous neuronal loss in the progression of specific neurodegenerative conditions such as amyotrophic lateral sclerosis and peripheral demyelinating diseases.^11^

To establish the most relevant molecular cascades triggered during the early stages of this dynamic degenerative process we have combined quantitative proteomics with morphological analysis and manipulation of nerves throughout the degenerative process *in vivo*.

Here we demonstrate that axonal injury initiates an early response in Schwann cells (SCs) resulting in fragmentation of their associated axons by actin-rich cytoplasmic spirals of SCs known as Schmidt-Lanterman incisures (SLI). Due to the molecular and mechanistic alterations we identify in this process which overlap with cellular cytokinesis, we term this process ‘axokinesis’. Importantly, interfering with axokinesis delays the course of mitochondrial-dependent degeneration of axons in a non-autonomous manner. Thus, in addition to their roles in myelin removal and macrophage recruitment in the later stages of axon degeneration, SCs are also early cellular participants of axon degeneration.

By detailing the complexity of this process, regulated by both neuronal intrinsic and extrinsic factors, we propose that a holistic *in vivo* approach will be required to more effectively control the degenerative process.

## Results

To study the process of axonal degeneration *in vivo* we have employed an injury induced degenerative paradigm, corresponding to a mechanical injury to the sciatic nerve *in vivo*.^12^ This system has many advantages over disease-induced degenerative models including the control of initiation site and timing; rapid and reproducible degenerative profile and reduced molecular noise.

### Early axonal fragmentation: the dynamics of myelin-ovoid formation

**Figure 1:**
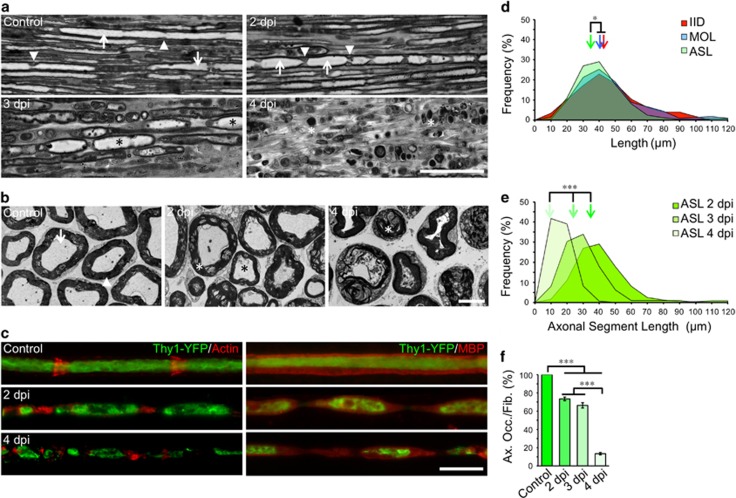
Dynamics of myelin-ovoid formation and axonal fragmentation. (**a**) Micrographs of semithin sections from longitudinally oriented nerves from WT mice. Intact (control) and distal nerves at 2, 3 and 4 days post injury (dpi) are shown. In control nerves, axons are clearly visible (arrows) and SLI are distinguishable (arrowheads). At 2 dpi axons appears discontinuous and myelin ovoids start to develop. At 3 dpi myelin ovoids encasing axonal segments are clearly identified (black asterisks) and at 4 dpi only myelin debris are distinguish (white asterisks). Scale bar, 50 *μ*m. (**b**) Electron micrograph from transverse sections of intact nerves (control) showing intact myelins (arrowhead) around axons (arrow). At 2 dpi the myelin is disorganized (white asterisk) and the axoplam is less defined (black asterisk) and by 4 dpi myelin sheaths are collapsed and axons degenerated. Scale bar, 5 *μ*m. (**c**) Teased fibers from Thy1-YFP mice stained for actin with rhodamine-conjugated phalloidin (red) in the left panels and immunostained against myelin basic protein (MBP, red) in the right panels. At 2 dpi, axons (green) appear fragmented and encased in a myelin ovoid and by 4 dpi axonal fragments are significantly reduced in size. Scale bar, 20 *μ*m. (**d**) Frequency distribution of inter incisure distance (IID), myelin ovoids length (MOL) and axonal segment length (ASL) measured from teased fibers at 2 dpi from WT mice. Color-coded arrows indicate the mean value in each set of data (*n*=3 mice per group, between 150 and 250 fibers were analyzed for IID, MOL and ASL; *P<0.05 by Student’s *t*-test compared with 2 dpi vehicle). (**e**) ASL distribution at 2, 3 and 4 dpi A significant shift toward lower values takes place with time (*n*=3 per group; *P<0.05 by one-way ANOVA plus Bonferroni *post hoc* test; error bars indicate S.E.M.). (**f**) Percentage of axonal occupancy per fiber (Ax. Occ/Fib.) measured from teased fibers (*n*=3 mice per group, 25 fibers per group; *P<0.05 by Student’s *t*-test compared with control or 3 dpi; error bars indicate S.E.M.)

In longitudinal sections of uninjured nerves (Figure 1a), SLI formed by SCs span the depth of the myelin. In nerves distal to a crush injury, the appearance of these SLI changes markedly. At 2 and 3 days post-injury (dpi), SLI are larger and protrude into the axon creating an axonal segment encapsulated within the inter incisure myelin fragment (termed an ‘ovoid’ Figure 1a). In transverse sections at 2 dpi, the compact myelin becomes delaminated, the axoplam disorganizes, mitochondria swell, and by 4 dpi myelin fills the former axonal space (Figure 1b).

#### In WT nerves axon fragments are contained within myelin ovoids

Employing fluorescence microscopy, a precise spatial correspondence between SLI and axonal fragmentation points is found at 2 dpi (Figure 1c). The distribution of inter-incisure distance (IID) and myelin ovoid length were comparable at 2 dpi (Figures 1d). When compared to IID, the distribution of axonal segments length (ASL) was shifted to smaller values, suggesting that these segments are contained within myelin ovoids (Figure 1d), shortening with time (Figure 1e). The axonal occupancy per fiber was evaluated as a measurement of axonal disintegration, decreasing with time after injury (Figure 1f).

The number of macrophages slightly alters at 2 dpi but are not associated with SLI (Supplementary Figures 1a and b). Fibroblasts do not increase in number even at 4 dpi, nor they associate to SLI (Supplementary Figure 1c).

#### Wld^s^ fragmentation process appears similar to WT but with delayed onset

**Figure 2:**
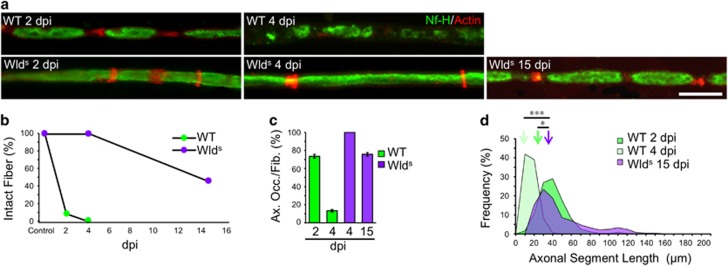
Axonal fragmentation and disintegration is delayed in Wld^s^ mice. (**a**) Teased fibers from WT mice sciatic nerves immunostained for neurofilament (Nf-H, green) and stained for actin filaments using phalloidin (red). At 4 dpi, Wld^s^ mice look like an uninjured nerve, but fragmentation began at 15 dpi Scale bar, 20 *μ*m. (**b**) Quantification of the percentage of unfragmented fibers (intact fibers) from teased fibers as shown in (**a**). In Wld^s^ there are not fragmented fibers at 4 dpi compared with WT mice, where all the fibers are fragmented at this time. At 15 dpi, half of the fibers are still intact in Wld^s^. (*n*=3 mice per group; between 80 and 100 fibers per group). (**c**) Percentage of axonal occupancy per fiber (Ax. Occ/Fib.) measured from teased fibers in WT and Wld^s^ nerves. At 15 dpi start the disintegration of axons in Wld^s^ (*n*=3 mice per group; 30 fibers per group were analyzed). (**d**) ASL distribution at 2 and 4 dpi from WT and 15 dpi from Wld^s^. Color-coded arrows indicate the mean value in each set of data (*n*=3 mice per group; between 90 and 120 ALS were measured per group; *P<0.05 by Student’s *t*-test compared with 2 or 4 dpi WT)

Given that Wld^s^ is thought to delay the normal degenerative process^13^ we next wanted to establish if this apparently glial mediated fragmentation process is also delayed. To this end, sciatic nerves of WT and Wld^s^ mice were injured and changes evaluated by immunofluorescence (IF). In WT nerves, axons associated with myelin ovoids disintegrate with time (Figure 1f), and become almost completely undetectable by 4 dpi (Figures 1c and f and Figures 2a–d). In sharp contrast, in Wld^s^ axons this degenerating process only starts around 15 dpi (Figures 2a–d), developing a fragmented profile comparable to those of WT nerves (Figure 2d), and indicating a similar process of fragmentation, but with a greatly delayed initiation.

These results therefore suggest that before axoplasm disintegration occurs, axonal injury elicits an early response from SCs associated with the fragmentation of axons by SLI.

### Axonal disintegration is regulated by mPTP activation in axons and can be genetically uncoupled from SC-dependent fragmentation

**Figure 3:**
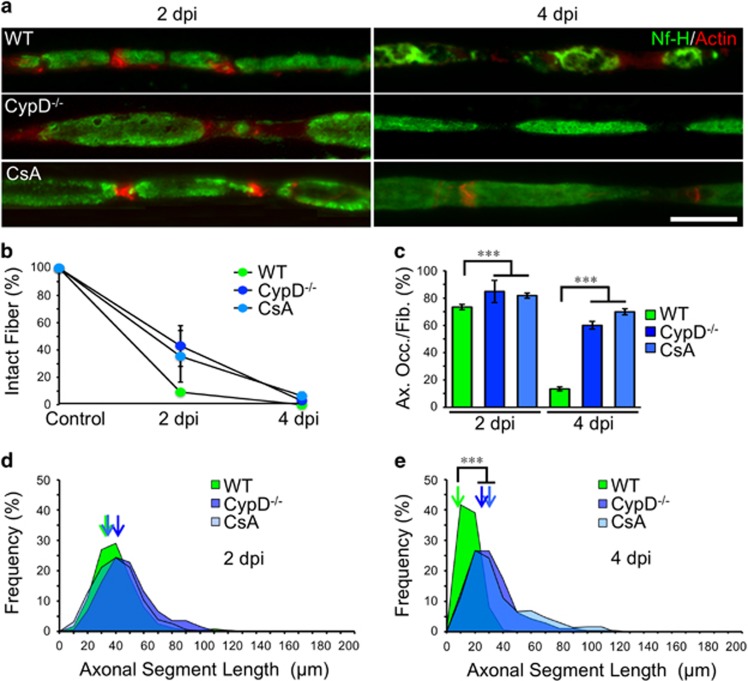
Axonal fragmentation by SC is mPTP-independent. (**a**) Teased fibers from WT, CypD^−/−^ and WT nerves injected with Cyclosporine A (CsA, 5 *μ*M) immunostained for neurofilament (Nf-H, green) and stained for actin filaments using phalloidin (red). At 4 dpi, axonal desintegration in CypD^−/−^ or CsA-treated nerves is inhibited compared to WT nerves. Axonal fragmentation is not altered by the genetic or pharmacological interventions. Scale bar, 20 *μ*m. (**b**) Quantification of the percentage of unfragmented fibers (intact fibers) from teased fibers as shown in **a**. There are no differences at 2 or 4 dpi in the percentage of fragmented fibers in CypD^−/−^ or CsA nerves (*n*=3 mice per group; 70 fibers per group; *P<0.05 by Student’s *t*-test compared with 2 or 4 dpi vehicle; error bars indicate S.E.M.). (**c**) Percentage of axonal occupancy per fiber (Ax. Occ/Fib.) measured from teased fibers in WT, CypD^−/−^ mice and CsA-injected nerves. At 4 dpi, the disintegration of axons is significantly inhibited in both cases (*n*=3 mice per group; 25 fibers per group; *P<0.05 by Student’s *t*-test compared with 2 or 4 dpi vehicle; error bars indicate S.E.M.). (**d** and **e**) ASL distribution at 2 and 4 dpi from WT, CypD^−/−^ and CsA-injected nerves. At 2 dpi all conditions are comparable, but at 4 dpi CypD^−/−^ and CsA-injected nerves have an ASL distribution shifted to higher values. Color-coded arrows indicate the mean value in each set of data (*n*=3 mice per group; between 80 and 180 ASL were analyzed per group; *P<0.05 by Student’s *t*-test compared with 2 or 4 dpi vehicle)

We have previously demonstrated that genetic inhibition of mPTP activation by cyclophilin D (CypD) knockdown delays axonal degeneration *in vitro*,^5^ therefore corresponding to an axonal autonomous process. At 2 dpi, axonal fragmentation in nerves from CypD^−/−^ mice was comparable to those of control mice (Figures 3a and b), as also was the ASL distribution (Figure 3d). Nevertheless at 4 dpi, axonal desintegration was strongly inhibited in CypD^−/−^ when compared to WT nerves (Figures 3c and e). Intraneural administration of cyclosporin A (CsA), an mPTP inhibitor, produced an effect comparable to that observed in CypD^−/−^ (Figures 3a–e). In addition, axonal mitochondrial swelling at 4 dpi – a reporter of the mPTP activation^14^ – was strongly inhibited in nerves of CypD^−/−^ mice or treated with CsA compared to degenerating nerves from WT mice (at 4 dpi: WT, 655.5±32.6 nm; CypD^−/−^, 276,1±23 nm; CsA, 345.5±21 nm, *P*<0.01 by Student’s *t*-test compared to WT).

We also studied the experimental nerves by electron microscopy (EM). In control, non-crushed nerves, well-defined axons were surrounded by an intact myelin sheath (Supplementary Figure 2). At 4 dpi, few axons remains and myelin sheets appear disorganized and collapsed (Supplementary Figure 2). In CypD^−/−^ nerves, axonal degeneration at 4 dpi was delayed. Some degenerated myelin were observed in CypD^−/−^ nerves at 4 dpi, that most likely corresponded to the poles of ovoids (Supplementary Figure 2).

Together, these data suggest that during the early stages of Wallerian degeneration axonal fragmentation by SCs is followed by a mPTP-dependent cell-autonomous process of axonal disintegration.

### Nerve degeneration is perturbed by pharmacological manipulation of SCs

#### Axonal fragmentation is delayed by nuclear program inhibition

**Figure 4:**
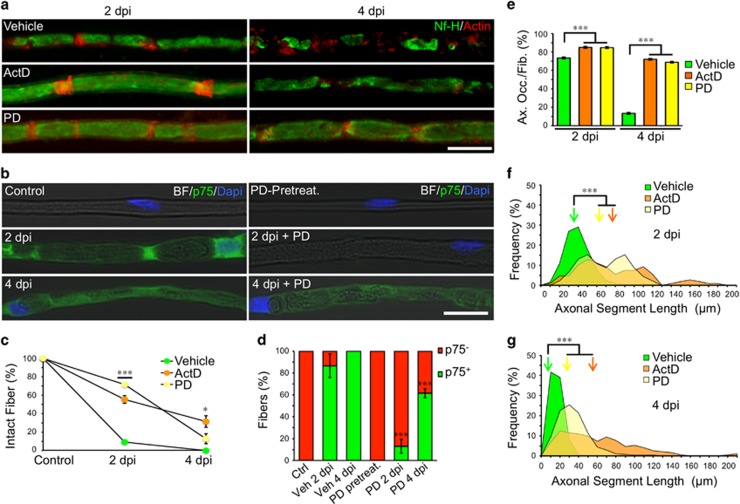
Schwann cells dedifferentiation is required for axonal fragmentation. (**a**) Teased fibers from WT sciatic nerves immunostained for neurofilament (Nf-H, green) and stained for actin filaments using phalloidin (red). Both Actinomycin D (ActD, 0.2 *μ*g/*μ*l) and PD 0325901 (PD, 2 *μ*M) treatments inhibits axonal fragmentation at 2 dpi and axonal disintegration at 4 dpi Scale bar, 20 *μ*m. (**b**) Teased fibers from vehicle or PD 0325901 (PD, 2 *μ*M) injected nerves, in light contrast microscopy (Bright Field, BF) immunostained with p75^NTR^ (green) and nuclear staining (Dapi) from control nerves and at 2 and 4 dpi In non-injured nerves (control and PD-pretreatment), Schwann cells (SC) are negative for the differentiated marker p75. In control nerves, SC express p75 at 2 and 4 dpi Injured-induced p75 expression is inhibited by PD treatment. Scale bar, 20 *μ*m. (**c**) Quantification of the percentage of unfragmented fibers (intact fibers) after each treatment from teased fibers as shown in (**a**). At 2 dpi both ActD and PD treatments significantly inhibit fiber fragmentation, while at 4 dpi, only ActD-treated nerves still have a significantly number of intact fibers (*n*=3 mice per group; between 60 and 80 fibers per group were counted; *P<0.05 by Student’s *t*-test compared with 2 or 4 dpi vehicle; error bars indicate S.E.M.). (**e**) Percentage of axonal occupancy per fiber (Ax. Occ/Fib.) measured from teased fibers in vehicle injected nerves and after ActD and PD injection. At 4 dpi, the disintegration of axons is significantly inhibited after drug treatments (*n*=3 mice per group; 25 fibers per group; *P<0.05 by Student’s *t*-test compared with 2 or 4 dpi vehicle; error bars indicate S.E.M.). (**d**) Quantification of the proportion of p75^NTR^-positive and -negative expressing fibers (p75^+^ or p75^-^) from teased fibers as shown in **b**. In non-injured fibers, all Schwann cells are p75^-^. At 2 dpi most Vehicle-injected fibers become p75^+^ (80%) and all of them express p75 at 4 dpi In PD-injected nerves, p75 expression is highly inhibited at 2 dpi (13%) and also at 4 dpi (60%), being statistically different from their controls (*n*=3 mice per group; between 30 and 50 fibers were analyzed per group; *P<0.05 by Student’s *t*-test compared with 2 or 4 dpi vehicle). (**f** and **g**) ASL distribution at 2 and 4 dpi from vehicle, ActD and PD-injected nerves. Drug treatments inhibit the reduction of ASL after injury. Color-coded arrows indicate the mean value in each set of data (*n*=3 mice per group; between 90 and 130 ASL per group; *P<0.05 by Student’s *t*-test compared with 2 or 4 dpi vehicle)

The role of SC genetic programs in Wallerian degeneration was studied with actinomycin D (ActD), which arrests transcription.^15^ Six days after an endoneurial injection of ActD, the sciatic nerve was crushed proximal to the injection site and the distal stump was analyzed. In control nerves, about 90% of fibers were fragmented at 2 dpi and completed by 4 dpi (Figures 4a and c). After ActD treatment, only 40% had undergone fragmentation at 2 dpi, and by 4 dpi, there was still unfragmented axons (Figures 4a and c). In addition, ActD treatment also affected the disintegration of axons at 4 dpi (Figure 4e). This is evident as a shift in the axonal segment distribution at both 2 and 4 dpi (Figures 4f and g). In ActD-treated nerves, EM observation reveals that axonal degeneration was delayed at 4 dpi (Supplementary Figure 2).

Axonal fragmentation is therefore delayed by days when transcription in SCs have been turned off, consequently affecting axonal disintegration.

#### Axonal fragmentation is delayed by inhibiting schwann cell dedifferentiation

After nerve injury, SCs dedifferentiate into a repair phenotype regulated by the ERK signaling pathway.^16^ As axonal fragmentation corresponds to an early event after nerve damage, we wanted to test if this process was dependent on SC dedifferentiation. We therefore blocked injury-induced SC dedifferentiation *in vivo* using the ERK inhibitor PD0325901. At 2 dpi, the expression of p75^NTR^, indicative of SC dedifferentiation, was not detectable following ERK inhibition (Figures 4b and d). At 2 dpi axonal fragmentation was delayed (Figures 4c and f) and axonal disintegration at 4 dpi was also delayed by ERK-inhibition (Figure 4e).

### Quantitative proteomic comparison between injured WT and Wld^s^ nerves reveals cytoskeletal changes correlating with axonal fragmentation

**Figure 5:**
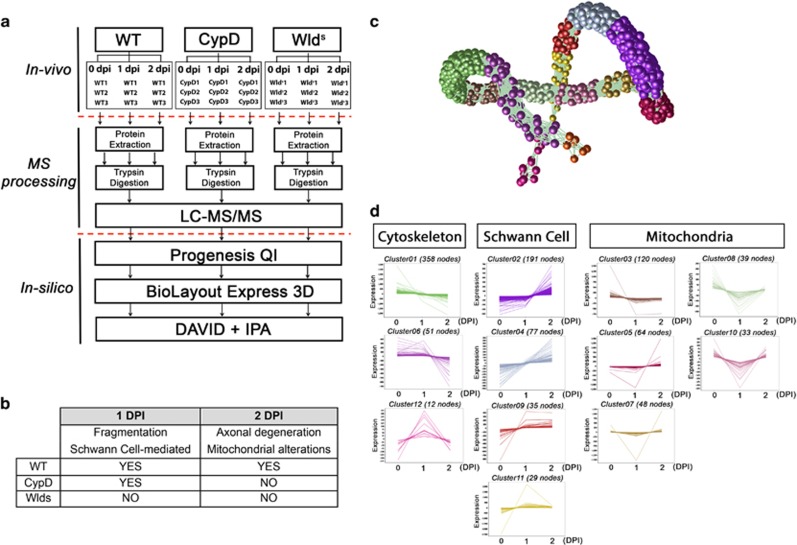
Temporal profiling of the molecular events underpinning injury induced nerve degeneration. (**a**) Workflow of proteomic analysis and processing of data. (**b**) Table illustrating expected morphological hallmarks which can be used as a filtering guide for proteomic data throughout the time-course examined. (**c**) BioLayout expression analysis allows a 3D representation of the molecular alterations occurring throughout the time course of WT degeneration. Each sphere represents an individual protein and its color and proximity to its neighbor indicates the similarity in protein expression (through time). Clusters (groupings of proteins delineated by color) can be further analyzed for cell type or functional association with other *in silico* tools such as DAVID thereby allowing the data to be broken down into more manageable groupings (see Materials and Methods). Example trends and functional categorizations can be seen in (**d**). The molecular response mapping through axonal degeneration correlated with morphological analysis can be used to elucidate relative contribution of specific cell types and organelles

We employed a quantitative proteomic approach to elucidate the molecular mechanisms governing the degenerative program. Nerve extracts were examined from multiple mouse lines at early stages of injury-induced degeneration (1 and 2 dpi, Figure 5a and Supplementary Table 1) and morphological correlates (Figures 1 and 2) were used as a filtering guide to determine the contribution of different processes (see Figure 5b and Methods).

First, we wanted to understand the molecular changes across the time course of nerve degeneration in WT mice. Figures 5c and d show a BioLayout generated schematic of WT time course data where clusters of co-expressed proteins are grouped in different colors. A DAVID enrichment analysis of clusters indicates that co-expressed proteins group into specific functional categories corresponding to cytoskeletal organization, mitochondria stability/function, and Schwann-cell-mediated responses (Figure 5d). This broad range of functional categories further highlights the complexity of the molecular cascades underpinning the degenerative process.

#### In-silico analysis at 1 dpi highlights ROCK as a potential focal regulator of early molecular cascades

**Figure 6:**
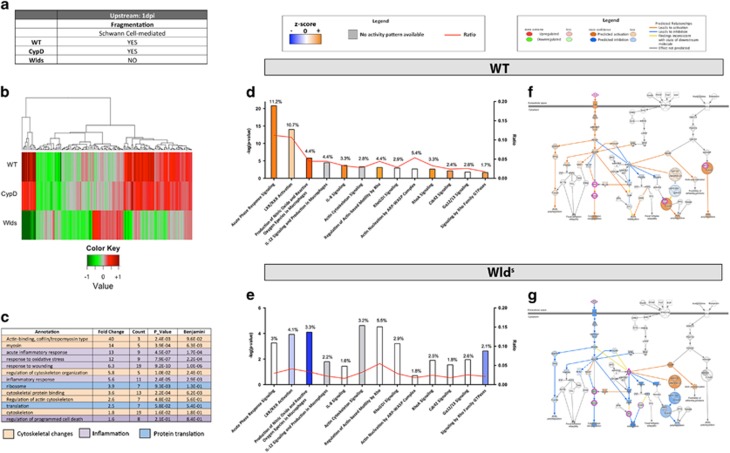
Bioinformatic analysis of proteomic data at 1 dpi reveals disruption of cytoskeletal related cascades. (**a**) Table illustrating expected morphological hallmarks that can be used as a filtering guide at 1 dpi (**b**) Heat map highlighting expression trends following filtering criteria at 1 dpi, that is, similar trends between WT and CypD^−/−^ but different in Wld^s^. (**c**) DAVID enrichment analysis of candidates following filtering highlights three main categories; cytoskeletal organization, inflammatory responses and protein translation which correlate with the known structural alterations present in WT nerves time-course analysis (Figures 4c and d). (**d** and **e**) Canonical pathway comparison between WT and Wld^s^ filtered candidates at 1 dpi highlights perturbations in cytoskeletal maintenance and inflammatory responses. Canonical pathways are ranked according to *P* value and the potential activation or suppression of that pathway is indicated by color (see Materials and Methods section – IPA prediction tools). Note that pathways such as acute phase response are activated in WT nerves but inhibited in Wld^s^. (**f** and **g**) Schematic representations of the actin cytoskeleton signaling cascade at 1 dpi and its expression/activation in WT and Wld^s^ nerves (respectively). Note that candidates such as ROCK, PFN, myosin and ARP are upregulated in WT promoting cytoskeletal re-organization relative to Wld^s^ nerves

After characterizing the molecular changes taking place in WTs we next wanted to identify factors associated with axonal fragmentation. Further filtering was applied to enrich for proteins related to the fragmentation mechanisms based on the differential degenerative responses of the mutant mouse nerves analysed. A total of 154 proteins were selected for *in silico* processing as they presented conserved protein expression between WT and CypD^−/−^, but differed to Wld^s^ at 1 dpi (Supplementary Figure 3a, Figures 6a and b and Supplementary Table 2).

Enrichment analysis of these candidates was carried out to identify which gross functions may be over represented in this group. DAVID identified functional annotations related to cytoskeletal regulation, inflammatory responses, and protein translation (Figure 6c). Knowing that there is no infiltration of macrophages at 1 dpi,^17, 18^ the molecular machinery identified as associating with inflammatory responses and protein translation pathways are more likely to be associated with SC activation following nerve damage.

Next, pathway analysis was carried out to identify specific cascades correlating with axonal fragmentation. For this, Ingenuity Pathway Analysis software (IPA) (Ingenuity Systems, Inc., Redwood City, CA, USA) was employed to compare differentially expressed proteins between WT and Wld^s^ mice at 1 dpi. The main altered pathways correlated with the enrichment analysis (Figure 6c), highlighting again that cytoskeletal regulatory cascades are prominently perturbed (Figures 6d and e). Specifically, cytoskeletal regulatory pathways are predicted to be activated in WT nerves, but inhibited in Wld^s^ nerves (Figures 6d and e). ‘Actin cytoskeletal signaling’, displayed in Figures 6f and g highlights the molecular changes associated with the activation of ROCK and myosin pathways regulating cytoskeletal reorganization. Profilin and ARP2/3 were also upregulated in WT nerves leading to actin polymerization (Supplementary Figure 4 and Supplementary Figure 5), while in contrast, the same pathways in Wld^s^ appear to be inactivated with ROCK described as inhibited, thereby reducing actin polymerization and cytoskeleton reorganizational processes in Wld^s^ nerves.

Other inflammatory-like response pathways related to the SC were detected. This correlates with the previous DAVID enrichment analysis as highly prominent in WT and not activated in Wld^s^ (Supplementary Figure 6).

This combined proteomic and bioinformatics analysis of WT and Wld^s^ peripheral nerves at 1 dpi therefore implicates cytoskeletal signaling pathways as potential regulators of axonal fragmentation.

### Axonal fragmentation by SCs operates using a cytokinesis-like process

#### Actin polymerization is required for axonal fragmentation but not disintegration

**Figure 7:**
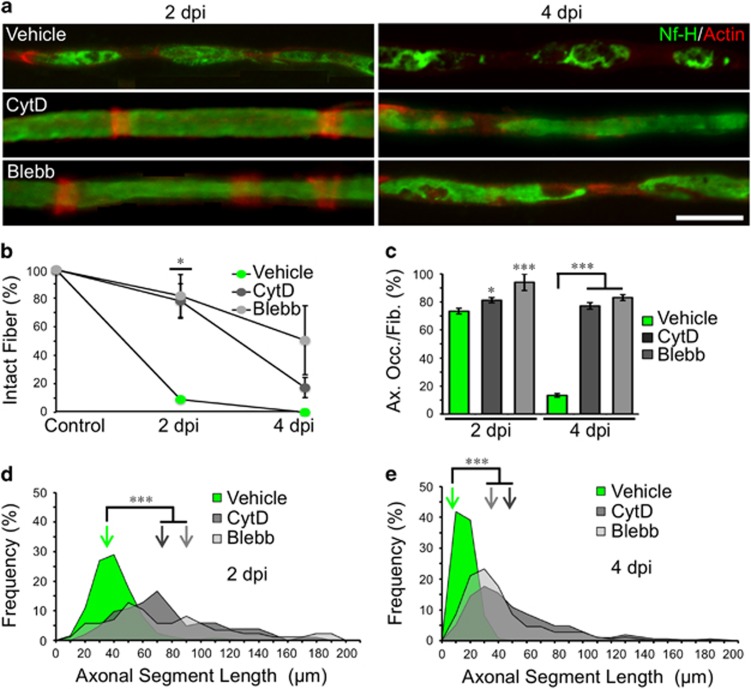
Axonal fragmentation is dependent on actin and myosin dynamics. (**a**) Teased fibers from WT mice sciatic nerves immunostained for neurofilament (Nf-H, green) and stained for actin filaments using phalloidin (red). Both Cytochalasin D (CytD, 2 *μ*g/ml) and Blebbistatin (Blebb, 500 *μ*M) treatments inhibits axonal fragmentation at 2 dpi and axonal disintegration at 4 dpi Scale bar, 20 *μ*m. (**b**) Quantification of the percentage of unfragmented fibers (intact fibers) after each treatment from teased fibers as shown in (**a**). Only at 2 dpi, both CytD and Blebb treatment significantly inhibit fiber fragmentation (*n*=3 mice per group; between 80 and 100 fibers per group; *P<0.05 by Student’s *t*-test compared with 2 or 4 dpi vehicle; error bars indicate S.E.M.). (**c**) Percentage of axonal occupancy per fiber (Ax. Occ/Fib.) measured from teased fibers in vehicle injected nerves and after CytD and Blebb injection. At 4 dpi, the disintegration of axons is significantly inhibited after drug treatments (*n*=3 mice per group; 30 fibers per group were analyzed; *P<0.05 by Student’s *t*-test compared with 2 or 4 dpi vehicle; error bars indicate S.E.M.). (**d** and **e**) ASL distribution at 2 and 4 dpi from vehicle, CytD and Blebb-injected nerves. Drug treatments inhibit the reduction of ASL after injury. Color-coded arrows indicate the mean value in each set of data (*n*=3 mice per group; between 90 and 120 ALS were measured per group; *P<0.05 by Student’s *t*-test compared with 2 or 4 dpi vehicle)

Our proteomic analysis comparing WT versus Wld^s^ mice, revealed major changes associated with cytoskeleton remodeling. As Schwann cell SLI are actin-rich structures,^19^ we explore whether actin dynamics were required for axonal fragmentation. To this end, we crushed WT sciatic nerves and injected Cytochalasin-D (CytD, a drug that inhibits actin polymerization) distal to the crush site. In CytD-treated fibers, axonal fragmentation was greatly reduced at 2 dpi (Figures 7a–d). At 4 dpi, the percentage of fragmented fibers was comparable between vehicle and CytD-treated nerves (Figure 7b), but axonal desintegration was delayed by CytD treatment (Figures 7c and e). This observation supports the proteomic data above.

#### Non muscle myosin II is required for axonal fragmentation and subsequent disintegration

Axonal fragmentation by SLI appears to be a process of actin-dependent constriction of the myelin sheath and axonal fragmentation, similar to the actomyosin contractile ring during cytokinesis.^20^ Cytokinesis involves two major scaffolds proteins: septins, that binds myosin II and anillin that links F-actin to the plasma membrane.^21^ In a control nerve, Myosin II is present in SLI and accumulates in ovoids tips after injury (Supplementary Figure 7). To explore whether axonal fragmentation uses similar molecular mechanisms as cytokinesis, we crushed nerves and inhibited non-muscle myosin II with Blebbistatin (Blebb). After myosin II inhibition we observed a significant reduction in fragmentation at 2 dpi (Figures 7a–d), and axonal disintegration was inhibited at 2 and 4 dpi (Figure 7c). EM observation reveals that at 4 dpi, Wallerian degeneration was delayed by Blebbistatin treatment (Supplementary Figure 2).

#### Anillin is required for axonal fragmentation by SCs

**Figure 8:**
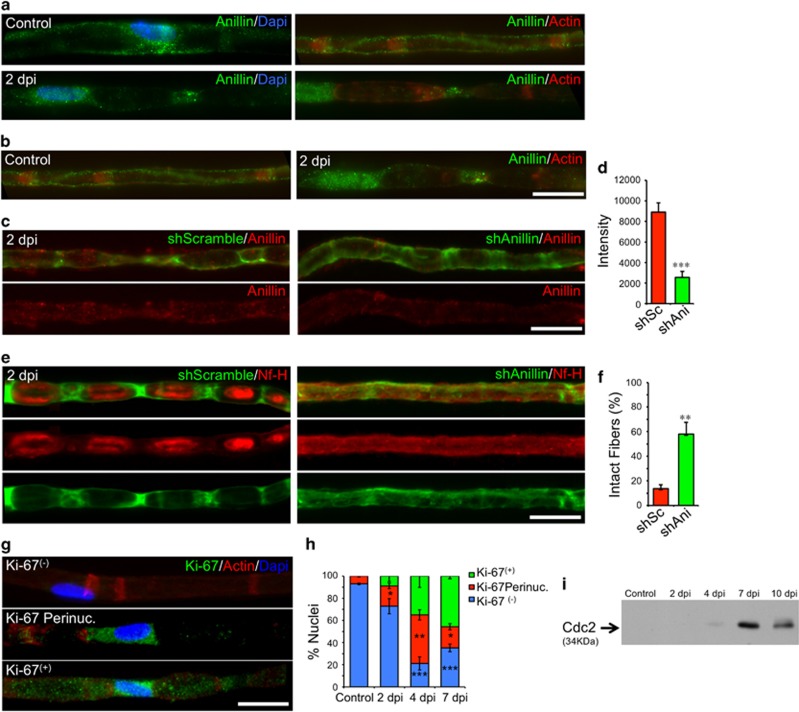
Schwann cell fragment axons by a cytokinesis-like mechanism. (**a**) Teased fibers from WT mice sciatic nerves from control conditions and at 2 dpi, immunostained for Anillin (green), actin (using rhodamine-conjugated phalloidin, red) and nuclei (using Dapi, blue). In control conditions, Anillin is located in the peri-nuclear region (right panel), along the SC cytoplasm known as Cajal bands. At 2 dpi, Anillin is expressed in the nucleus and at the ends of myelin ovoids. Scale bar, 20 *μ*m. (**c**) Teased fibers from shRNA-electroporated nerves at 2 dpi GFP (green) expression is a reporter for electroporation. Control shRNA-treated nerves (shScramble, left panel) or shAnillin (right panel) immunostained for Anillin (red). Scale bar, 20 *μ*m. (**d**) Quantification of Anillin expression by immunofluorescent intensity in teased fibers electroporated with the different constructs. A significant decrease in protein expression is found in SC electroporated with shAnillin (shAni) compared with shScramble-electroporated fibers (shSc, *n*=5 mice per group; 30 fibers per group; *P<0.05 by Student’s *t*-test compared with shScramble; error bars indicate S.E.M.). (**e**) Teased fibers from nerves electroporated with shScramble (green, left panel) or shAnillin (green, right panel) and co-stained with neurofilament (Nf-H, red). Anillin knockdown strongly inhibit axonal fragmentation compared with control fibers. Scale bar, 20 *μ*m. (**f**) Quantification of axonal fragmentation at 2 dpi in nerves electroporated with the indicated constructs (*n*=5 per group; 30 fibers per group; *P<0.05 by Student’s *t*-test compared with shScramble; error bars indicate S.E.M.). (**g**) Teased fibers immunostained for Ki67 (green), actin (red) and nuclei (blue) showing the absence or the different subcellular localization of Ki67 expression. (**h**) Quantification of Ki67 localization from teased fiber as in panel G. Ki67 expression is absent in control and 2 dpi fibers, to start in a peri-nuclear sub-localization at 2 dpi and 4 dpi and in a nuclear localization at 7 dpi (*n*=3 mice per group; between 80 and 100 fibers per group; *P<0.05 by Student’s *t*-test compared with control; error bars indicate S.E.M.). (**i**) Cdc2 protein expression at different times after nerve injury. Protein expression begins at 4 dpi and reaches a peak at 7 dpi

Other important molecular players in cytokinesis are septin 2 and anillin. Septin 2 has been observed at SC nodes of Ranvier, Cajal Bands and SLI.^22^ In control conditions, we observed anillin expression in the perinuclear region of SCs and along Cajal Bands (Figures 8a and b). At 2 dpi, anillin was present in the nucleus and at the poles of the ovoids (Figures 8a and b), a change in distribution analogous to the one that takes place during cytokinesis.^23^ We used an shRNA strategy to deplete anillin from SCs *in vivo* by local nerve electroporation (see Materials and Methods). This procedure significantly decreases anillin expression in SCs (Figures 8c and d). We then assessed the fragmented fibers in nerves electroporated with a scramble or anillin shRNA at 2 dpi using a fluorescent reporter for electroporated SCs. In SCs treated with the scramble shRNA, non-fragmented axons were <20% at 2 dpi whereas treatment with anillin-shRNA resulted in a three-fold inhibition of axonal fragmentation by SCs (Figures 8e and f).

#### Axonal fragmentation does not coincide with SC proliferation

We next assessed whether this cytokinesis-like mechanism of axonal fragmentation was dissociated from Schwann cell proliferation, a process that usually takes place at 3 to 4 dpi in WT nerves.^24^ To this end, we analyzed the expression and localization of the protein Ki-67 which translocates into the nuclei of dividing cells (reporter for G1 to M phase transition). Localization of Ki-67 was perinuclear at 2 dpi and nuclear at 4 dpi (Figures 8g and h). In addition, Western blot analysis of Cdc2 expression, a protein associated to G2/M phases, was undetected at 2 dpi, and strongly upregulated at 7 dpi (Figure 8i). Together, this data suggest that axonal fragmentation by Schwann cells is not associated to Schwann cell proliferation after nerve injury.

#### ROCK activity is required for axonal fragmentation

**Figure 9:**
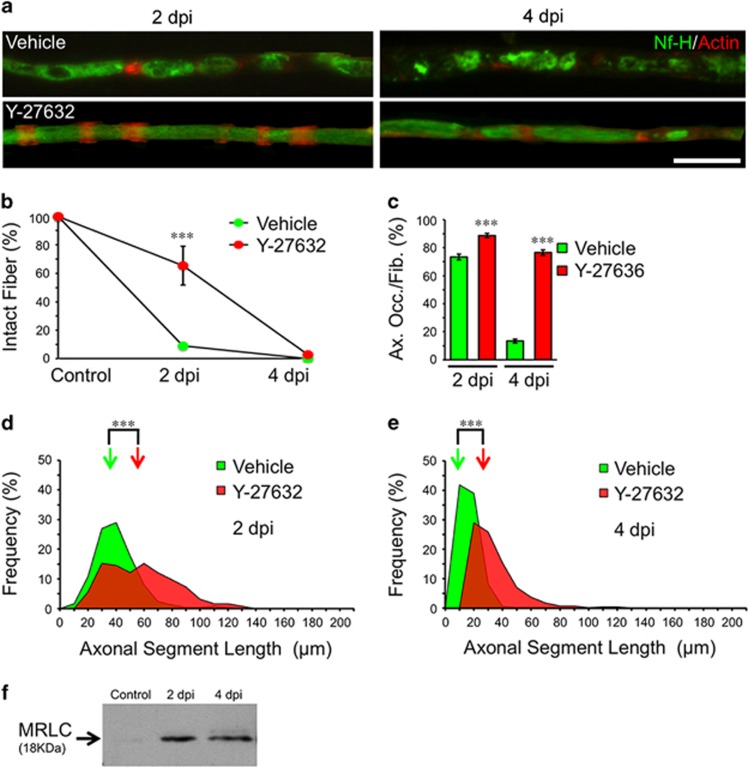
Axonal fragmentation is ROCK dependent. (**a**) Teased fibers from WT mice sciatic nerves immunostained for neurofilament (Nf-H, green) and stained for actin filaments using phalloidin (red). Y-27632 (50 *μ*M) treatment inhibits axonal fragmentation at 2 dpi and axonal disintegration at 4 dpi Scale bar, 20 *μ*m. (**b**) Quantification of the percentage of unfragmented fibers (intact fibers) after treatment from teased fibers as shown in (**a**). Only at 2 dpi, Y-27632 treatments significantly inhibit fiber fragmentation (*n*=3 mice per group; between 80 and 100 fibers per group; *P<0.05 by Student’s *t*-test compared with 2 or 4 dpi vehicle; error bars indicate S.E.M.). (**c**) Percentage of axonal occupancy per fiber (Ax. Occ/Fib.) measured from teased fibers in vehicle-injected nerves and after Y-27632 injection. At 4 dpi, the disintegration of axons is significantly inhibited after drug treatments (*n*=3 mice per group; 30 fibers per group were analyzed; *P<0.05 by Student’s *t*-test compared with 2 or 4 dpi vehicle; error bars indicate S.E.M.). (**d** and **e**) ASL distribution at 2 and 4 dpi from vehicle and Y-27632-injected nerves. Drug treatment inhibits the reduction of ASL after injury. Color-coded arrows indicate the mean value in each set of data (*n*=3 mice per group; between 90 and 120 ALS were measured per group; *P<0.05 by Student’s *t*-test compared with 2 or 4 dpi vehicle). (**f**) MRLC protein expression at different times after nerve injury

During cytokinesis, phosphorylation of the myosin related light chain (MRLC) by ROCK regulates the interaction of actin and myosin, leading to the formation of the contractile ring.^25^ Therefore, we pharmacologically inhibited the kinase function of ROCK *in vivo*. To this end, the ROCK inhibitor Y-27632 was injected into the nerve and crushed proximal to the injected region. At 2 dpi, axonal fragmentation was strongly inhibited by Y-27632 compared to vehicle injected and crushed nerves (Figures 9a–e). In addition, both at 2 and 4 dpi, axonal occupancy and axonal segment length were significantly higher in Y-27632-injected nerves compared to vehicle-treated ones (Figures 9c–e). Importantly, MRLC phosphorylation was detected at early time points after nerve injury by Western blot (Figure 9f). This is consistent with the predicted regulators identified by our proteomic experiments.

#### Axonal fragmentation *in vitro* occurs independently of SC mechanisms identified above

As *in vivo* injections targets Schwann cells and axons, we tested the effect of all used drugs in axons devoid of Schwann cells *in vitro* (Supplementary Figure 8a). In all cases, mechanically induced axonal degeneration was not modified by ActD, PD0325901, CytD, Blebb or Y-27632 (Supplementary Figures 8b and c), suggesting that *in vivo* these drugs have a direct effect over Schwann cells that impact the progression of axonal fragmentation.

### Axonal fragmentation by SCs is independent of mitochondrial dysfunction in the axonal compartment

The axon has internal programs for self-destruction that involve mitochondria, in particular the activation of the mPTP,^5^ triggered by release of ER-derived calcium, leading to ROS production, and secondary activation of proteases.^4, 13^ Since SCs fragments the axon before its disintegration, we studied the relationship between axonal fragmentation and the activation of the axonal destruction cascade by analyzing the CypD^−/−^, in which the activation of the mPTP is inhibited.^26^

#### Axonal-dependent degenerative processes correlate with perturbations in 3 metabolic cascades

**Figure 10:**
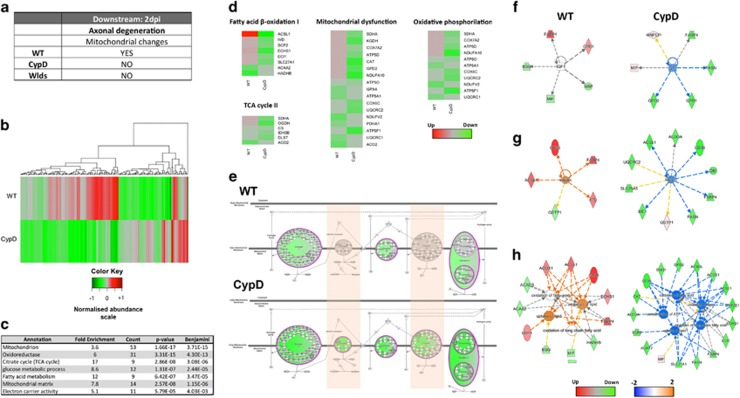
Bioinformatic analysis of proteomic data at 2 dpi reveals molecular perturbations correlating with the intrinsic axonal mediated destruction cascades previously reported. (**a**) Table illustrating expected morphological hallmarks, which can be used as a filtering guide at 2 dpi (**b**) Heat map highlighting expression trends following filtering criteria at 2 dpi, that is differential expression between WT and CypD^−/−^ (**c**) DAVID enrichment analysis of candidates following filtering highlights mitochondria and bioenergetics cascades which correlate with the known structural alterations present in WT nerves at 2 dpi (**d**,**e**) Canonical pathway alterations correlate to DAVID enrichment and highlight downregulation in a broad range of mitochondrial associated metabolic processes which are in agreement with published data suggesting that factors such as cytochrome *c* release from complex IV is inhibited in CypD^−/−^ vs WT nerved. (**f**–**h**) Upstream regulator predictions and biofunction analysis suggest the inhibition of IGF, insulin and oxidative metabolic regulated processes (respectively) in CypD^−/−^ relative to WT nerves at 2 dpi

In order to elucidate the mechanisms related to mitochondrial-dependent degeneration in axons, we employed a variation on the previous described proteomic filtering strategy (Figure 10a). We focused on 2 dpi as axonal disintegration occurs after fragmentation has already begun (Figures 1 and 2). We identified 190 proteins differentially expressed between WT and CypD^−/−^ mice at 2 dpi and overlapping with mitochondrial clustering (Figures 5c and d, Figure 10b,Supplementary Figure 3, and Supplementary Table 3).

A DAVID enrichment analysis highlighted pathways related to mitochondria oxidative and metabolic cascades such as the TCA cycle, glucose or fatty acid metabolism (Figure 10c). Next, IPA analysis confirmed the perturbation of multiple canonical pathways including mitochondrial dysfuction, oxidative phosphorylation, TCA and fatty acid oxidation – i.e., perturbation of normal mitochondrial cascades (Figure 10d).

#### Constituents from all the mitochondrial inner membrane complexes are differentially expressed in CypD nerves at 2 dpi

In terms of oxidative phosphorylation there is a trend toward modest downregulation of constituents from complex I, III and IV in WT nerves (Figure 10e), which contrast to a strong downregulation of members from all five mitochondrial inner membrane complexes in CypD^−/−^, specially complex IV. Interestingly, complex IV is responsible for the release of cytochrome *c* – a process that has been reported to be blocked in CypD^−/−^.^27^ Importantly, reduction in mitochondrial complex activity, including IV, has been associated with a reduction in ROS production,^28^ an important factor in axonal degeneration.^12^

#### Pathway-based predictions identify specific metabolic factors as potential regulators for the identified pertubations in CypD degenerating nerves

An analysis for potential upstream regulators highlighted the inhibition of IGF-1, insulin and restriction of metabolic cascades (that is, fatty acids and lipid processing) in CypD^−/−^ relative to WT expression levels (Figures 10f and g, respectively) and this has previously been described as contributing to axonal neuroprotection.^12^

This combined proteomic and bioinformatic analysis of WT and CypD^−/−^peripheral nerves at 2 dpi therefore implicate specific mitochondrial candidates as mediators of axonal-dependent disintegration, suggesting that axonal fragmentation and disintegration corresponds to dissectible degenerating pathways in the glia-axonal unit.

## Discussion

Axon degeneration can be initiated by a broad range of triggers including, but not limited to injury, toxic and genetic insults, and is an important early event in many pathological conditions.^29, 30^ Crucially, effective axonal degeneration is essential for subsequent regeneration and functional recovery after nerve damage.^31^ Yet, despite the importance of axon degeneration in injury, disease and regeneration, very little is known about the factors that govern the process.

Here we have attempted to address this by taking a novel multi-model *in vivo* approach, which combined morphological assesment and quantitative proteomics alongside *in silico*-based higher order functional clustering.

Mechanistically, we have found that after nerve injury, SCs fragment the axon by a mechanism analogous to cytokinesis which we term ‘axokinesis’ (Figures 7 and 8 and Supplementary Figure 9). Interfering with axokinesis affects the course of axonal disintegration in a non-autonomous way. Based on our results, we propose that the ERK cascade – critical for SC dedifferentiation^16^ – is the controlling program involved in axonal fragmentation. Importantly, delayed axonal degeneration of Wld^s^ axons is followed by delayed SC-dependent fragmentation, suggesting that an axonal autonomous role of the Wld^s^ protein may delay the intercellular signaling event that instructs SCs to dedifferentiate and fragment the axon. Studies on Wallerian degeneration are commonly performed from 4 dpi onwards, when axons are almost completely disintegrated and macrophages have started to invade the damaged nerve. By focusing on early time points after damage we report the identification of this novel process of axokinesis that regulates axonal-autonomous mechanisms of axonal degeneration.

A comparative analysis between WT and Wld^s^ revealed cytoskeletal-signaling perturbations correlating with axonal fragmentation processes taking place between 1 and 2 dpi (Figure 6) highlighting differential expression of key actin dynamic candidates activated in WT nerves during fragmentation and inactivated in its absence in the Wld^s^. Actin, myosin, anillin and ROCK are all instrumental in axonal fragmentation, since the interference of any of these actors by genetic or pharmacological means affects axonal fragmentation.

We conclude that SC participates actively in early steps of the disintegration of the axoplasm. That is, the axon has its own destruction mechanisms but SC can speed them up. Our comparative proteomic and bioinformatics approach examining CypD^−/−^ nerves where mPTP is inhibited (and axonal desintegration is delayed) at 2 dpi, correlated with the morphological data observed (Figure 3). The molecular data presented here therefore supports the notion that inhibition of several metabolic pathways collectively contribute to the neuroprotective effect demonstrated in CypD^−/−^ mice (Figures 10c–e).

Our unbiased approach identified multiple complex molecular cascades originating from non-neuronal autonomous glial cell processes, and then proceeding via the neuronal axon. This is a novel notion, as it is generally accepted that destruction of the axon is regulated by neuronal intrinsic processes. However, the data generated here point to a collaborative effort, a sort of cellular euthanasia. Moreover, these results suggest that activation of SC axokinesis could lead to axonal fragmentation, indirectly impacting neuronal function, a phenomenon that should be evaluated in neurodegenerative conditions, in which axonal degeneration has been demonstrated as an early event of the pathology.

## Materials and methods

### Animals

Adult (12 weeks, 20–25 grs) Ppif^−/−^ (CypD^−/−^) KO, Thy1-YFP, Wld^s^ and wild-type C57/BL6J mouse strains were used.^26, 32^ Experiments with animals followed protocols approved by the Institutional Animal Care and Use Committees and complied National Institutes of Health guidelines.

### Sciatic nerve injury and intraneural drug treatments

The animals were anesthetized with 2’2’2-tribromoethanol (Sigma, St. Louis, MO, USA, 330 mg/kg) and the right sciatic nerve was exposed between the hipbone and the Notch and crushed 3 times for 5 s each with a Nº5 Dumont forceps. In all conditions, the nerve was dissected 2 or 4 days post injury. For drug treatments, intraneural injections were made in the tibial fascicle of the sciatic nerve. For most drugs the treatment was performed daily starting from the day of crush. For ActD, the drug was injected 6 and 3 days before the nerve crush. The following drugs and concentrations were used: Actinomysin D (Calbiochem, San Diego, CA, USA, #114666, 0,2 *μ*g/*μ*l), PD 0325901 (Cayman, Ann Arbor, MI, USA, #13034, 2 *μ*M), Cytochalasin D (Sigma, #C6637, 2 *μ*g/ml), Blebbistatin (Sigma, #B0560, 500 *μ*M), Ciclosporin A (LC Laboratories, Woburn, MA, USA, #C6000, 5 *μ*M) and Y-27632 (Sigma, #Y0503, 50 *μ*M).

### Teased fiber IF

For IF analysis, nerves were fixed by immersion in 4% paraformaldehyde in 1 × PBS (pH 7.4) for 1 h, followed by three 10 min washes in 1 × PBS. Under a dissecting microscope, the perineurium was removed and fibers were teased in TESPA-coated slide and stored at −20 degrees. Teased fibers were placed directly into pre-chilled (−20ºC) acetone for 20 min at −20 ºC. After air dry the acetone, slides were blocked/permeabilized in 0.1% Triton X-100, 5% fish skin gelatin (Sigma, G7765) in 1 × PBS 1 h at room temperature (RT) and incubated overnight with primary antibodies in the same solution at 4 °C using a humid chamber. Next day fibers were washed in 1 × PBS three times 10 min and incubated in secondary antibodies for 2 hours at RT. Slides were washed 3 × 10 min in 1 × PBS and mounted in Fluoromount (EMS, Hatfield, PA, USA, #17984).

### Electron microscopy

For EM analyses, nerves were fixed overnight by immersion in 2.5% glutaraldehyde, 0.01% picric acid, 0.1 M cacodylate buffer, pH 7.4. Nerves were rinsed in the same buffer, immersed in 1% OsO_4_ for 1 h followed by in block incubation with 2% uranyl acetate for 2 hours. Nerves were dehydrated with a graded series of ethanol, propylene oxide and infiltrated with Epon (Ted Pella Inc. # 18012). One micron thick semithin sections from the middle of the nerve were stained with 1% toluidine blue for light microscopy. Ultra thin sections from the same nerve were contrasted with 1% uranyl acetate and lead citrate. Grids were examined with a Philips Tecnai 12 electron microscope operated at 80 kV.

### Primary/secondary antibodies and dyes

The following antibodies were used: rabbit anti-neurofilament heavy chain (Sigma, #N4142) at 1:1000, rabbit anti-myelin basic protein (Sigma, #M3821) at 1:500; rabbit anti-p75NTR (Millipore, #07-476) at 1:300; goat anti-anillin (Santa Cruz, #sc-54859) at 1:50; rabbit anti-myosin IIA (OneWorld, #bs_8564R) at 1:50; goat anti-rabbit Alexa Fluor 488 (Invitrogen, #A11034) at 1:1000; goat anti-rabbit Alexa Fluor 546 (Invitrogen, #A11035) at 1:1000; rabbit anti-goat Alexa Fluor 488 (Invitrogen, #A11078) at 1:1000 and Rhodamine-conjugated Phalloidin (Sigma, #P1951) at 1:1000.

### *In vivo* shRNA experiment

shRNA construct were purchased from Biosettia (San Diego, CA, USA). The following sequences for the Anillin gene were used: shAnillin-1: 5′-AAAAGCATCTGCTAGCATCAATATTGGATCCAATATTGATGCTAGCAGATGC-3′, shAnillin-2: 5′-AAAAGCAAGCATCTAGAAACCAATTGGATCCAATTGGTTTCTAGATGCTTGC-3′, shAnillin-3: 5′-AAAAGCGCTCAATATCTCTTCAATTGGATCCAATTGAAGAGATATTGAGCGC-3′, shAnillin-4:

5′-AAAAGCTCTGACATTTCCTACTATTGGATCCAATAGTAGGAAATGTCAGAGC-3′ and Scramble: 5′-AAAAGCTACACTATCGAGCAATTTTGGATCCAAAATTGCTCGATAGTGTAGC-3′. The constructs were amplified and purified with an AxiPrep Plasmid Miniprep Kit (Axygen, Union City, CA, USA, AP-MN-P-250), injected one microliter of the four shAnillin together (final concentration 0.73 *μ*g/*μ*l) or Scramble (0.72 *μ*g/*μ*l) in the nerve and then electroporated using a Super Electroporator NEPA 21 Type II (NepaGene, Chiba, Japan) with the following conditions: Poring pulse 50 V, 5 ms length, 50 ms interval, 2 pulses, 7 times per nerve. After 4 days, the injected nerves were damage and fibers analyzed 2 days post injury.

### DRG explant cultures, IF and analysis

Dorsal root ganglia (DRGs) were obtained from Sprague Dawley rat embryos (E16). Briefly, DRGs were dissected and placed on coverslips coated with rat tail collagen (Invitrogen). DRGs were maintained in Neurobasal medium (Invitrogen) supplemented with 2% B27 (Invitrogen), 2 mM L-glutamine, 50 ng/ml human nerve growth factor (NGF; Invitrogen) and 1% penicillin–streptomycin, 4 *μ*M Aphidicolin and 7.5 *μ*g/ml 5-fluoro-2- deoxyuridine. The mixture of aphidicolin and fluoro-2-deoxyuridine inhibits proliferation of Schwann cells by inhibition of DNA polymerase. DRGs were cultured for 7 d at 37 °C and 5% CO_2_. At day 7, ganglia were excised using a micropipette tip. This procedure eliminated all neuronal somas from the ganglia. The following drugs were used: Actinomycin D 1 ng/ml, PD0325901 0.1 *μ*M, Citochalasyn-D 1 *μ*g/ml, Blebbistatin 100 *μ*M and Y-27632 10 *μ*M. The degeneration index was based on the ratio of the areas of fragmented axons versus total axonal area. Degenerated axon fragments were detected using the particle analyzer algorithm of NIH ImageJ, and the total fragmented axon area versus total axonal area was used to estimate a degeneration index.

For IF analyses, DRGs were fixed with 4% PFA for 30 min at room temperature, followed by 2 h blocked/permeabilized in 1% Triton X-100, 5% fish skin gelatin (Sigma) in PBS. Cells were then stained with rabbit anti-neurofilament heavy chain (NFH; catalog #N4142; Sigma-Aldrich) at 1:1000 overnight at 4 °C, washed three times in PBS for 5 min, and incubated with fluorescently labeled secondary antibody (Alexa546; Life Technologies) for 2 h at room temperature. Cells were washed in PBS and mounted in Fluoromount.

### Data analysis and statistics

All slides were photographed in an epifluorescence Olympus microscope (Miami, FL, USA) and analyzed for the following parameters: Inter-incisure distance (IID), distance between two adjacent incisures positive for phalloidin; Myelin ovoid length (MOL) the distance between the beginning and the end of the ovoid, discriminated by bright field; axonal segment length (ALS), labeled with neurofilament and the percentage of fragmented fiber, quantification of intact versus fragmented fibers in bright field; axonal coverage per fiber, the ratio between the sum of the length of all the axonal segment in one fiber and the length fiber. Ten to 15 random pictures per slide were taken for each animal, time and treatment. All measurements were perform using the Image J software and calibrated microscope images. All the fibers in each pictures (from 2 to 15) were quantified for each measurements. Statistical analysis was made using Student’s *t*-test and one way ANOVA plus Bonferroni *post hoc* test.

### Label-free proteomics

The experimental design for label-free proteomics experiments was based on previous similar analyses^33, 34, 35^ and is shown in Figure 5a. Protein isolated from preparations of WT, CypD^−/−^ and Wld^s^ mouse sciatic nerve were extracted in SDT lysis buffer containing 100 mM Tris-HCl (pH 7.6), 4% (W/V) Sodium dodecyl sulfate (VWR) and 0.1 M d/l-dithiothreitol (Sigma). Each genotype was examined at three time points — 0, 1 and 2 dpi with 4 mice at each time point per condition. For efficient protein extraction, lysates homogenized in SDT buffer in a MACS Dissociator tube. Protein concentration was then determined using BCA assay and confirmed by total protein gel as previously described.^36^ For label-free proteomic analysis, single aliquots for each time point within each condition were prepared (100–25* μ*g per mouse) and were processed through FASP (filter-aided sample preparation) involving buffer exchange to 8 M urea and alkylation with 50 mM iodoacetamide prior to a double digestion with trypsin (Roche, sequencing grade), initially for 4 h, then overnight at 30 °C. Trypsin-digested peptides were separated using an Ultimate 3000 RSLC (Thermo Fisher Scientific, Waltham, MA, USA) nanoflow LC system. Using an ESI Easy Spray source at 50 °C, technical replicates (3 × ∼2.5 *μ*g) of each ‘pooled’ sample were loaded with a constant flow of 5 *μ*l/min onto an Acclaim PepMap100 nanoViper C18 trap column (100 *μ*m inner diameter, 2 cm length; Thermo Scientific). After trap enrichment, peptides were eluted onto an Acclaim PepMap RSLC nanoViper, C18 column (75 *μ*m, 50 cm; Thermo Scientific) with a linear gradient of 2–40% solvent B (80% acetonitrile with 0.08% formic acid) over 128 min with a constant flow of 300 nl/min. The HPLC system was coupled to a linear ion trap Orbitrap hybrid mass spectrometer (LTQ-Orbitrap Velos Pro, Thermo Scientific) via a nanoelectrospray ion source (Thermo Scientific). The spray voltage was set to 1.6 kV, and the temperature of the heated capillary was set to 250 °C. Full-scan MS survey spectra (m/z 335–1800) in profile mode were acquired in the Orbitrap with a resolution of 60,000 after accumulation of 1,000,000 ions. Lock mass was set at 445.120024. The 15 most intense peptide ions from the preview scan in the Orbitrap were fragmented by collision-induced dissociation (normalized collision energy, 35% activation Q, 0.250; and activation time, 10 ms) in the LTQ after the accumulation of 10,000 ions. Dynamic exclusion parameters were set as follows: repeat count, 1; repeat duration, 30 s; exclusion list size, 500; exclusion duration, 45 s; exclusion mass width, plus/minus 10 p.p.m. (relative to reference mass). Maximal filling times were 1000 ms for the full scans and 150 ms for the MS/MS scans. Precursor ion charge state screening was enabled, and all unassigned charge states as well as singly charged species were rejected. The lock mass option was enabled for survey scans to improve mass accuracy. Data were acquired using the Xcalibur software (Thermo Fisher Scientific, Waltham, MA, USA).

The raw data was then imported to Progenesis QI for label-free differential analysis and quantification of relative abundance ratios. A pool of all samples was run per triplicate as quality control samples (QC). Runs were aligned to a QC sample for the identification of common features. After the peak picking across all data files, identified peptides with *P* value >0.05 (one way ANOVA) and power<0.8 were filtered in order to reduce excess of unreliable data. Next, peptide information was exported and used for peptide sequence identification and protein assignment with Mascot using the Uniprot mouse database. Protein identifications were then imported back to Progenesis for further analysis and filtering. 47 418 search hits were imported and assigned to peptide ions, which was equivalent to a total of 1839 proteins. For further analysis, however, only proteins identified by ≥2 peptides and *P* value <0.05 were used (1067 proteins, Supplementary Table 1).

### Proteomic data processing

Due to the heterogeneity of the whole nerve cellular homogenates further filtering steps were required to unravel the contribution of each cell types to the molecular mechanisms of axonal fragmentation or axonal degeneration (Supplementary Figure 3).

Fragmentation was studied at 1 dpi (See Materials and Methods, Figure 5) and its mechanisms was unravel by enriching for proteins that are more likely to be associated with the early axonal fragmentation cascades (see Results and Figure 5b). As fragmentation occurs in both WT and CypD^−/−^ nerves, but not in Wld^s^ at early stages, proteins were selected, which demonstrate conserved protein expression alterations between WT and CypD^−/−^, but which differ in Wld^s^ at 1 dpi Specifically, we are seeking to elucidate the molecular cascades/immediate early responses occurring between 0 and 1 dpi in WT nerves and how expression in the CypD^−/−^ and Wld^s^ mutants compare in relation to those processes (i.e., relative to WT 1 dpi). Of the 1064 proteins identified in all samples at all time points, 154 proteins accomplish this criteria and were utilized in this section (Supplementary Figure 3A,Figure 6b and Supplementary Table 2).

Internal axonal mechanisms of destruction were studied at 2 dpi (See Figure 10). In order to enrich our samples for axonal dependent cascades rather than SC-associated ones (see above), we filtered the data for proteins displaying differential expression between WT and CypD^−/−^ nerve isolates and overlap with clusters related to mitochondria in the WT time-course analysis (Figures 5c and d). Of the 1064 proteins identified in all samples at all time points, 190 met this filtering criteria (Supplementary Figure 3B, Figure 10b and Supplementary Table 3).

### *In silico* analysis

#### BioLayout Express 3D

this software incorporates a complex pattern recognition algorithm, which allows grouping of data by expression profile independent of protein function or identification information. This software was developed in the Roslin Institute and further information on the use of this program and free access can be found in http://www.biolayout.org.

Analysis of WT proteomic data using biolayout allows clustering of protein abundance by temporal expression profile (Figures 5c and d). In this display each sphere represents an individual protein and its color and proximity to its neighbor indicates the similarity in protein expression (through time). Clusters (groupings of proteins delineated by color) can be further analyzed for cell type or functional association with other *in silico* tools – see below (Figure 5d).

### Enrichment analysis

To obtain an indication of the level of sample enrichment afforded the data was processed using The Database for Annotation, Visualization and Integrated Discovery (DAVID) software (available at http://david.abcc.ncifcrf.gov). David provides a relatively comprehensive set of functional annotation tools for large data set interpretation.^37, 38^

#### Ingenuity pathway analysis (IPA) software

This is a ‘hand–curated’ database that analyses and identifies statistically significant functional candidate associations, based on known interactions and biological functions reported in the published literature. This information is derived from peer reviewed publications and can be traced back to data at the genomic, transcriptomic and/or proteomic level. Functional categorization, function and/or predicted activations are therefore an indication of how data (in this case proteomic identifications and abundance) sits within the context of the known ‘interactome’. Canonical pathway tools were used for the identification of known pathways within these data sets. Pathways from IPA library of canonical pathways were ranked according to significance of the association between our data sets and the known canonical pathway members. Details about how these are calculated can be found in the published literature.^39^ Data imported into IPA were subjected to a 1.2-fold filter and searched against only those database entries, which were derived from experimentally observed data (i.e., not predicted/homology derived).

## Publisher's Note

Springer Nature remains neutral with regard to jurisdictional claims in published maps and institutional affiliations.
