# Supplementary figures and images for: Molecular analysis of axonal-intrinsic and glial-associated co-regulation of axon degeneration
Source: Cell Death Dis. 2017 Nov 9;8(11):e3166–. doi: 10.1038/cddis.2017.489 (PMC5775402; doi:10.1038/cddis.2017.489)

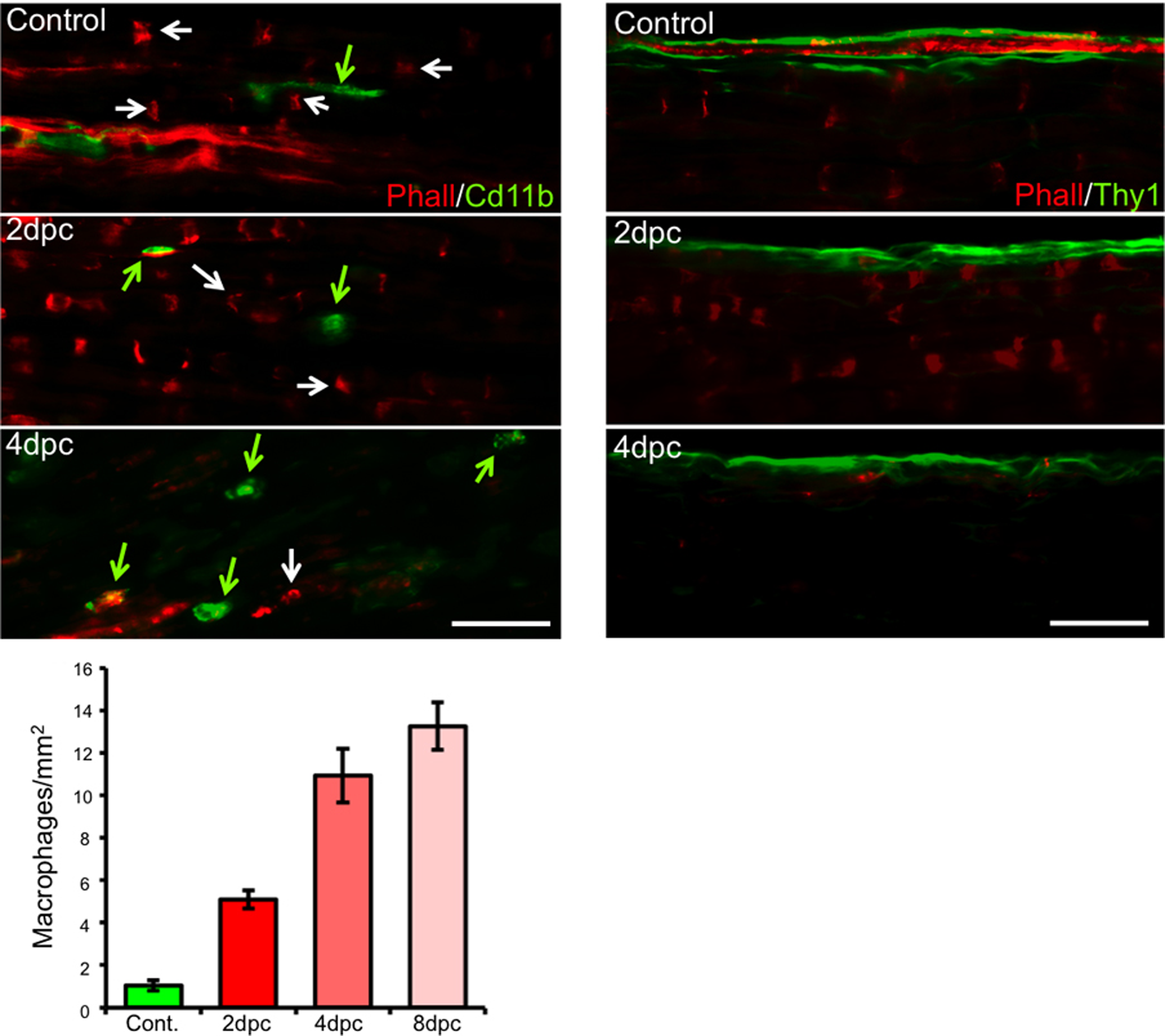

Supplement: Supplementary Figure 1 [file cddis2017489x1.tif]

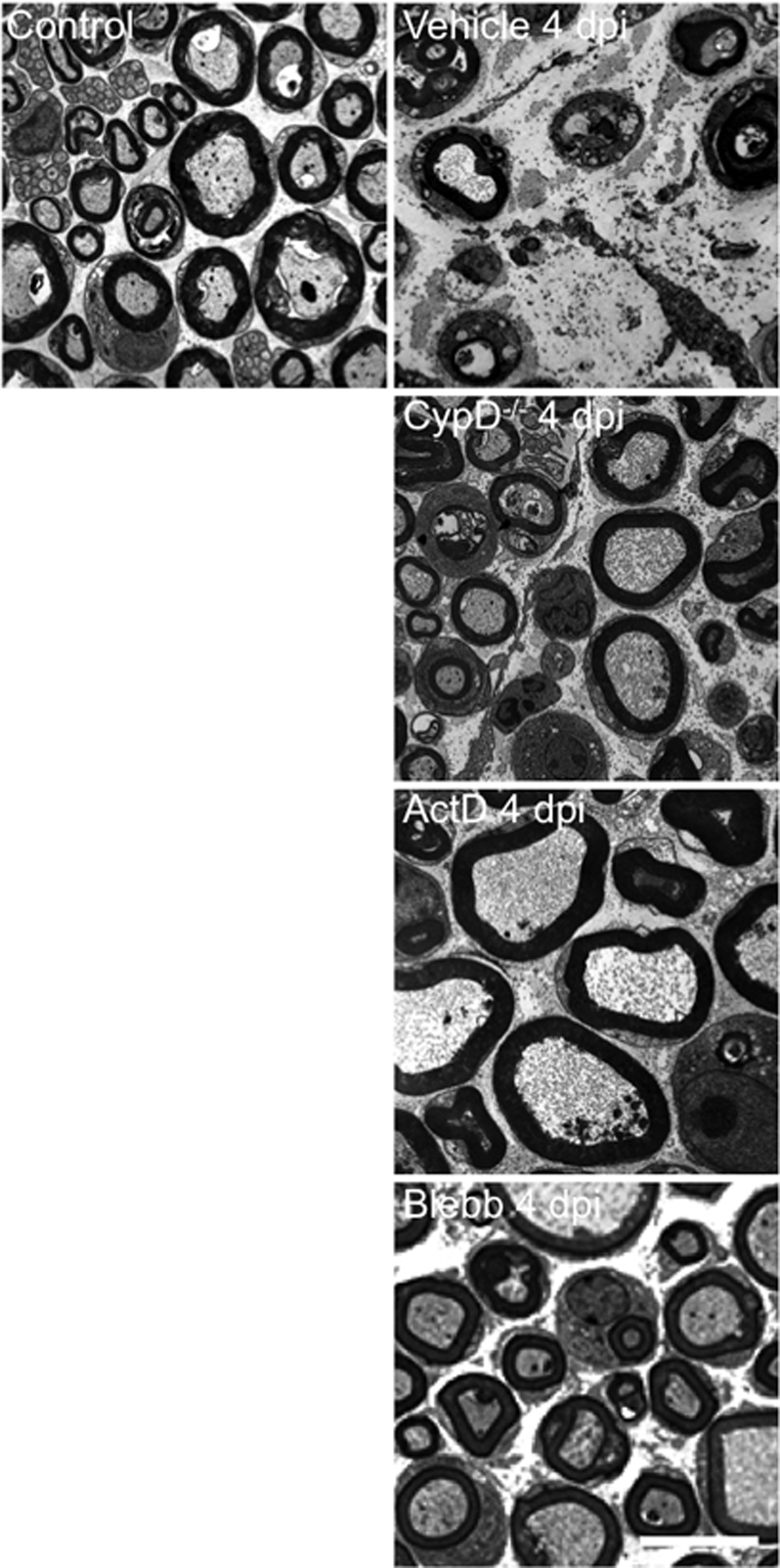

Supplement: Supplementary Figure 2 [file cddis2017489x2.tif]

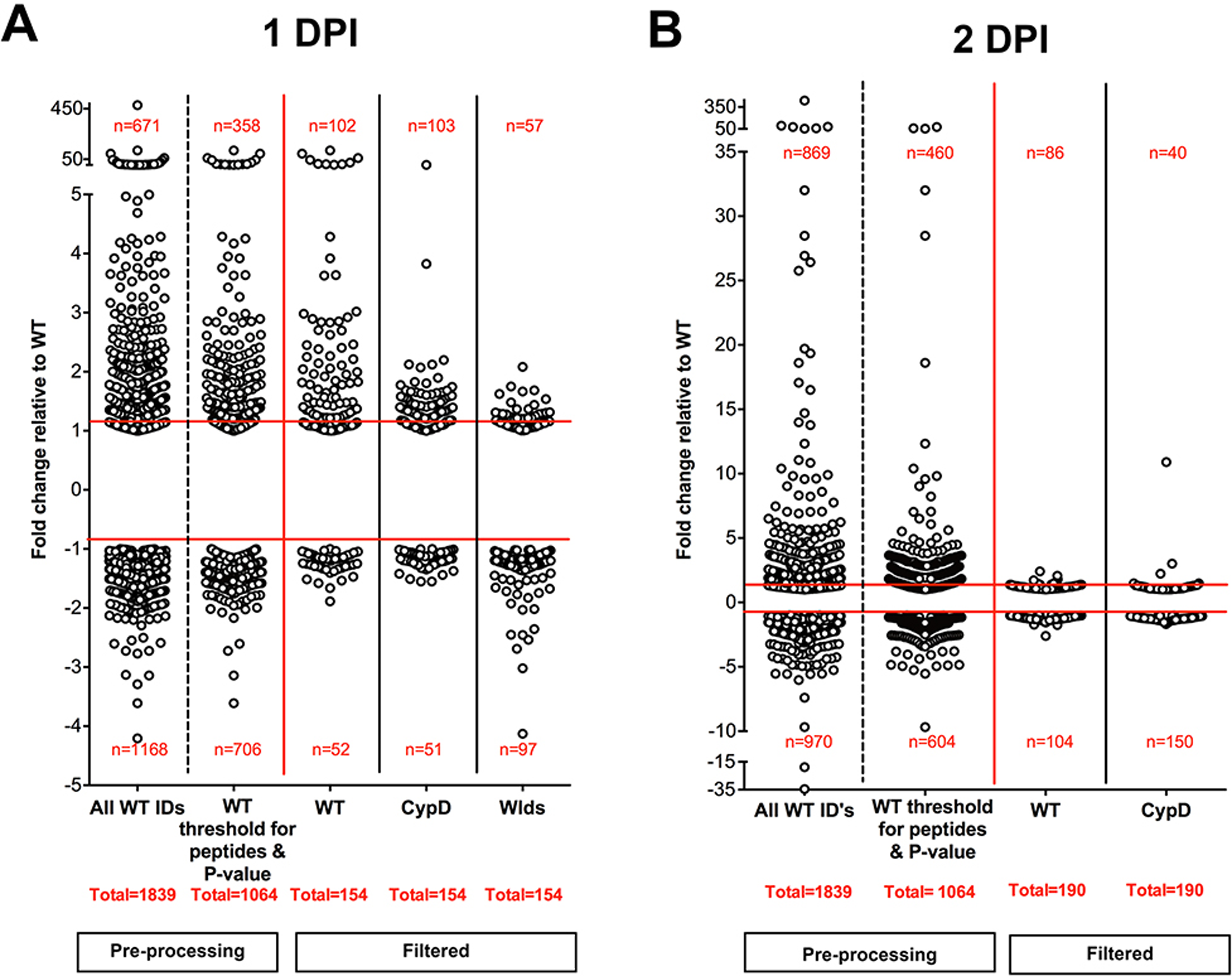

Supplement: Supplementary Figure 3 [file cddis2017489x3.tif]

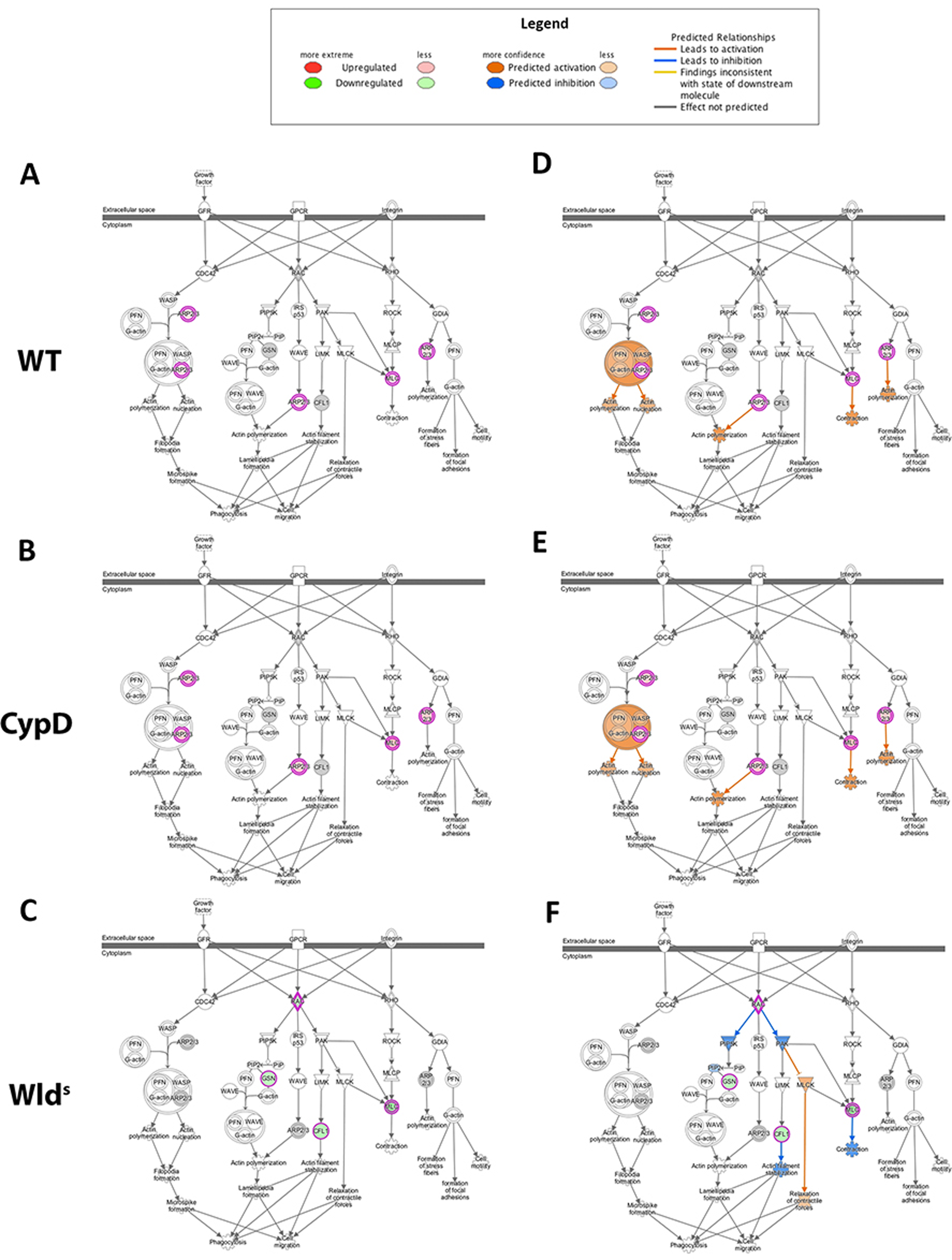

Supplement: Supplementary Figure 4 [file cddis2017489x4.tif]

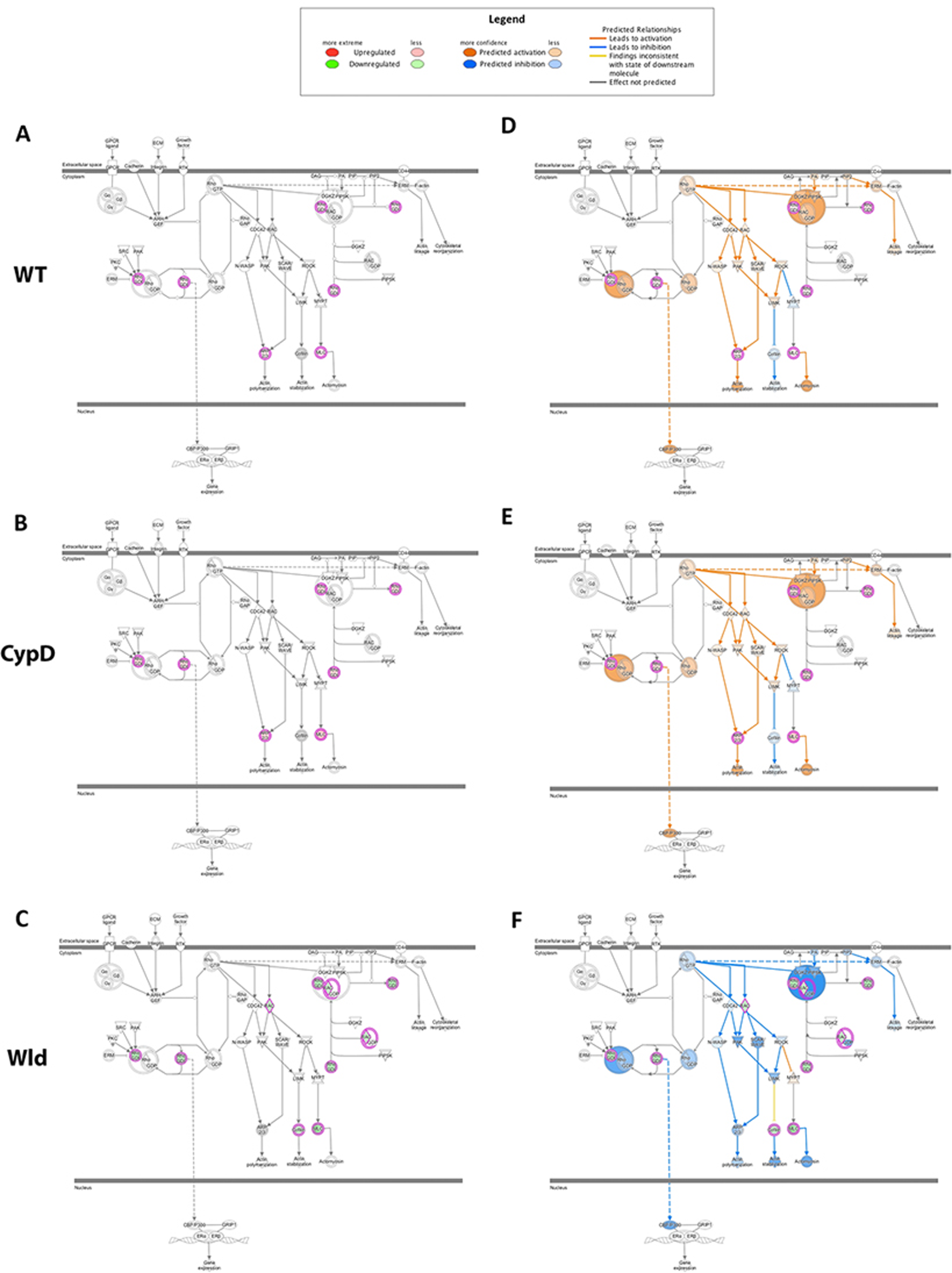

Supplement: Supplementary Figure 5 [file cddis2017489x5.tif]

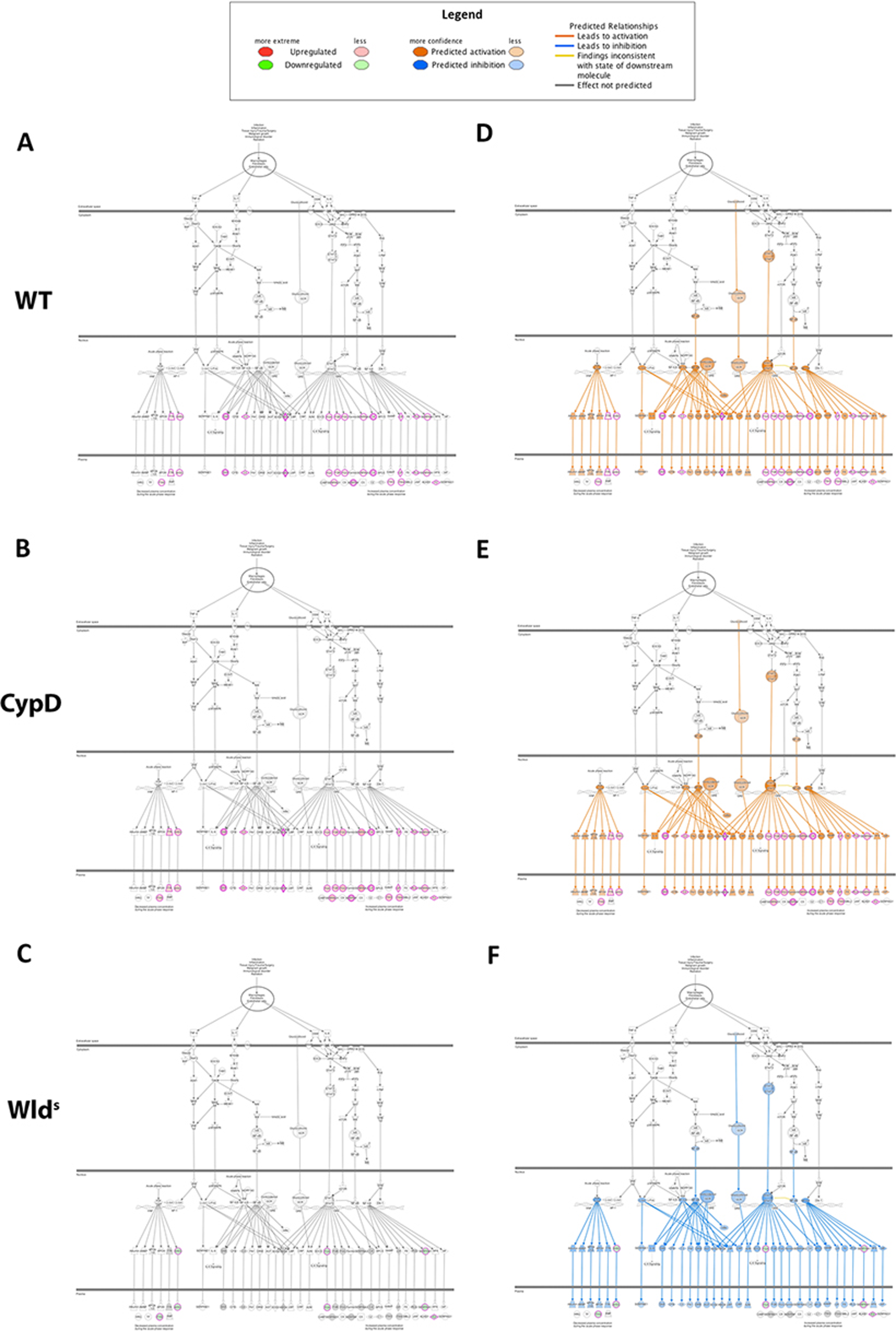

Supplement: Supplementary Figure 6 [file cddis2017489x6.tif]

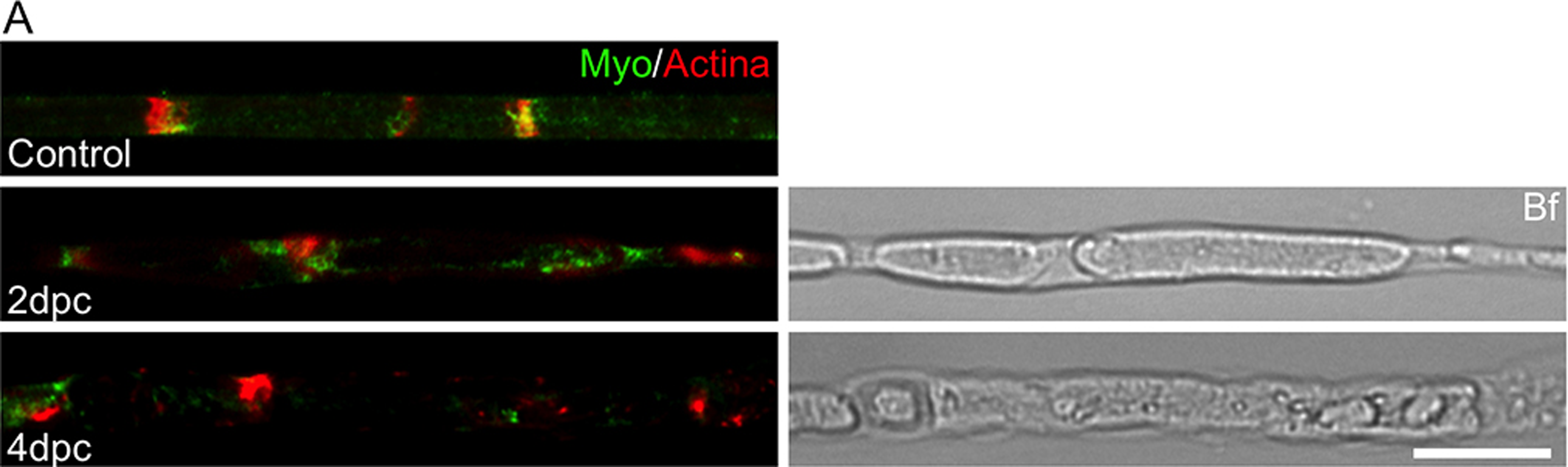

Supplement: Supplementary Figure 7 [file cddis2017489x7.tif]

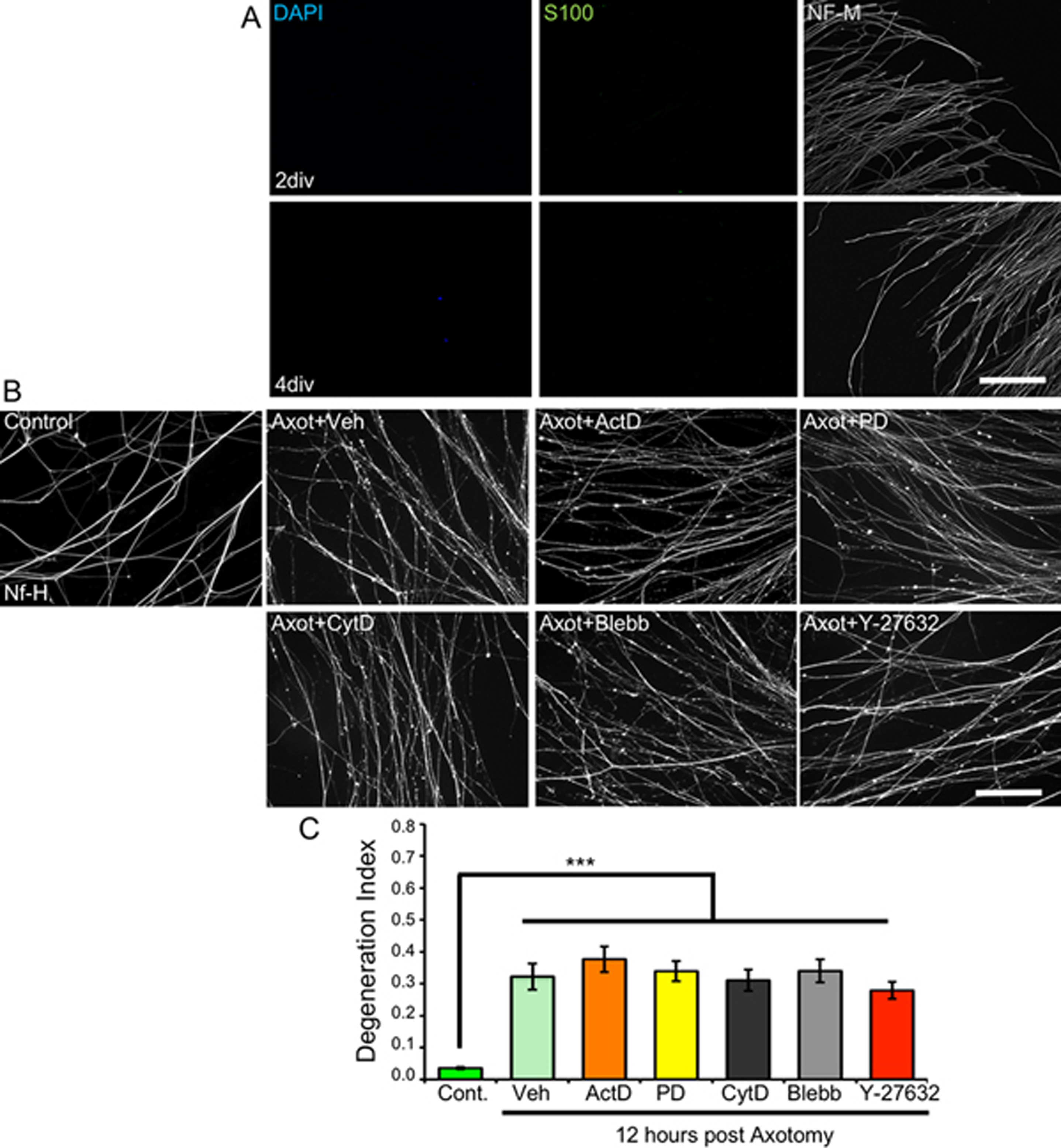

Supplement: Supplementary Figure 8 [file cddis2017489x8.tif]

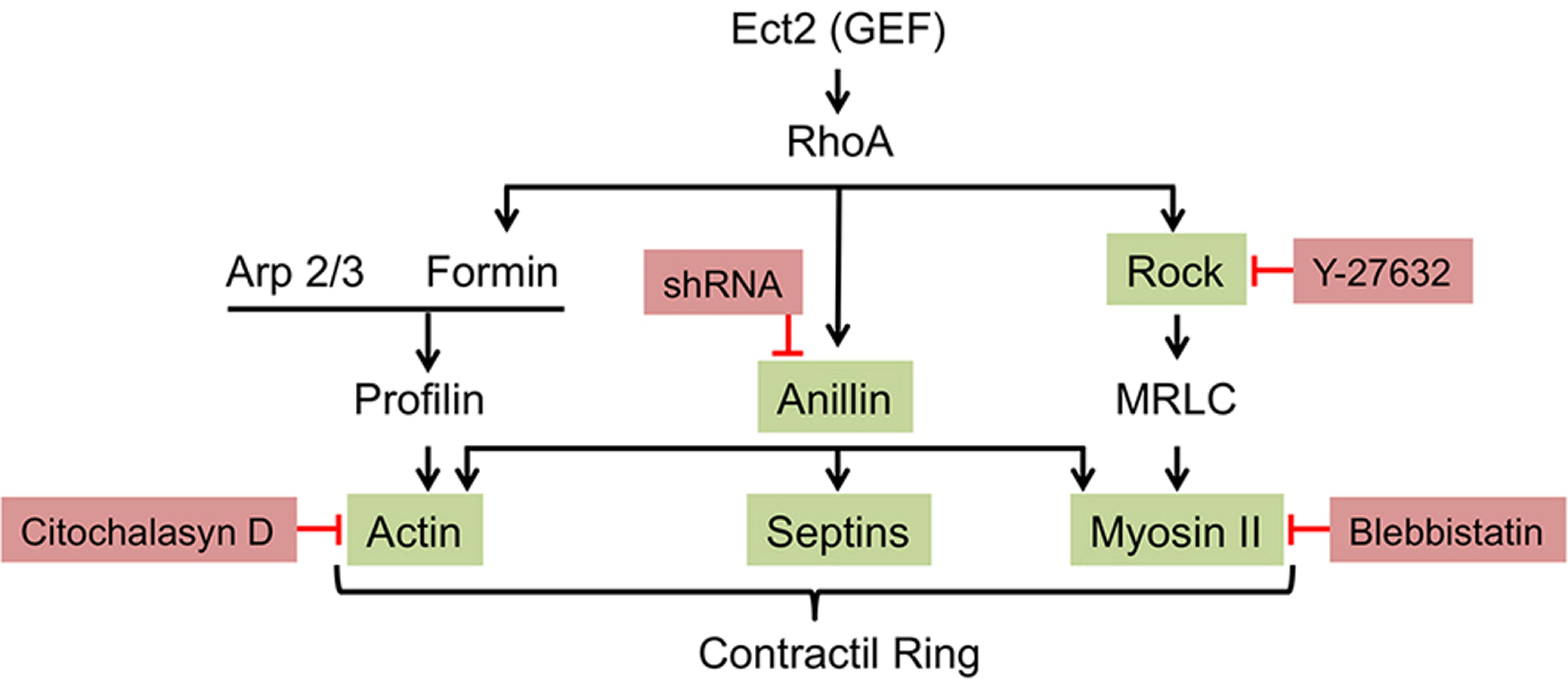

Supplement: Supplementary Figure 9 [file cddis2017489x9.tif]
