# Supplementary material for: Molecular analysis of axonal-intrinsic and glial-associated co-regulation of axon degeneration
Source: Cell Death Dis. 2017 Nov 9;8(11):e3166–. doi: 10.1038/cddis.2017.489 (PMC5775402; doi:10.1038/cddis.2017.489)
Supplement: Supplementary Figure Legends [file cddis2017489x13.docx]

**Supplementary Figure Legends**

**Supplementary Figure 1.** Macrophages and fibroblasts at early time points after nerve injury. **(A)** Immunostaining of macrophages using Cd11b (green arrows) and stained for actin filaments using phalloidin (red). At 2 dpi, actin-rich SLI can be seen in open or closed conformations and the few macrophages observed at 2 dpi, are not associated with SLI’s. **(B)** Macrophage numbers in the nerve increase with time after injury. **(C)** Immunostaining for fibroblasts using Thy-1 and stained for actin filaments using phalloidin (red). Scale bar, 25 µm**.**

**Supplementary Figure 2.** Electron micrographs from transverse sections of sciatic nerve. WT (Control), CypD^-/-^ ActD and Blebb-injected nerves were studied at 4 dpi. In the intact WT nerve, organized myelin around intact axons can be seen. At 4 dpi in Veh-injected nerves, myelin sheaths are collapsed and axons degenerated. In 4 dpi CypD^-/-^, ActD and Blebb-injected nerves, the tissue is not affected to the same extent and axons exhibit greater axoplasm preservation. Scale bar, 10 µm.

**Supplementary Figure 3.** Proteomic data filtering **(A-B).** Schematic of data filtering indicating the number of protein identifications after progeneisis analysis, and the subsequent candidate refinement following filtering criteria for 1 and 2 dpi, respectively (from Fig. 5B). At 1 dpi the comparison is change WT0 – WT1 vs CypD1 vs Wld^s^1 and at 2 dpi the comparison is change WT0 – WT2 vs CypD2. Please see results section 3 and 5 (respectively for more information).

**Supplementary Figure 4**. Regulation of actin-based motility by Rho is activated in WT and CypD^-/-^ nerves following injury **(A-C)** IPA schematic of “Regulation of actin-based motility by Rho” for WT, CypD^-/-^ and Wld^s^ nerves at 1 dpi. Key proteins such as ARP2/3 and MLC are upregulated in nerve undergoing fragmentation (WT and CypD^-/-^) and not in Wld^s^ which fragmentation is delayed until 15 dpi. **(D-F)** Same cascades with molecule activity prediction tool showing the activation of actin polymerization and contraction in WT and CypD^-/-^ and not in Wld^s^.

**Supplementary Figure 5**. Actin polymerization is activated in WT and CypD-/- nerves following injury **(A-C)** IPA schematic of “RhoGDI signaling” for WT, CypD^-/-^ and Wld^s^ nerves at 1 dpi. Key proteins such as ARP2/3 and RhoGDI are upregulated in nerve undergoing fragmentation (WT and CypD^-/-^ ) and not in Wld^s^ which fragmentation is delayed until 15 dpi. **(D-F)** Same cascades with molecule activity prediction tool showing the activation of actin polymerization and linkage as well actomyosin in WT and CypD^-/-^ and not in Wld^s^.

**Supplementary Figure 6**. Multiple inflammatory-related transcription factors are activated in WT and CypD-/- nerves following injury **(A-C)** IPA schematic of “Acute phase response signaling” in WT, CypD^-/-^ and Wld^s^ nerves at 1 dpi. Multiple proteins downstream the acute phase response cascade are upregulated in nerve undergoing fragmentation (WT and CypD^-/-^) but not in Wld^s^ which fragmentation is delayed until 15 dpi. Interestingly at 1 dpi there is no infiltration of macrophages yet, therefore these changes are likely to be triggered by SC activation instead. **(D-F)** The same cascades with molecule activity prediction tool showing the activation of multiple inflammatory-related transcription factors and other proteins. In contrast, this signaling cascades are inhibited in the Wld^s^ nerves.

**Supplementary Figure 7. Myosin distribution in Schwann cells. (A)** Teased fibers from sciatic nerves of WT mice from control, 2 dpi and 4 dpi conditions, immunostained for myosin II (green), actin (using rhodamine-conjugated phalloidin, red) and nuclei (using Dapi, blue), left panel. In control conditions, myosin II is located in SLI together with actin. At 2 dpi, myosin II is expressed at the ends of myelin ovoids (right panel) and a 4 dpi the expression of myosin II is decreased but still located at the ends of ovoids. Scale bar, 20 µm**.**

**Supplementary Figure 8. Drugs treatments do not delay axonal degeneration directly (A)** DRG explants cultured for seven days in the absence of Schwann cells. Axons are stained using Nf-H (green). In control condition (without axotomy) axons appears intact. At 12 hours post axotomy, degeneration of axons identified by discontinuities and swellings in the Nf-H staining is comparable in vehicle-treated cultures and after treatments with each drug (ActD, PD, CytD, Blebb and Y-27632). Scale Bar 100 µm. **(B)** Quantification of axonal degeneration (Degeneration Index) in control and after axotomy in vehicle (Veh) and ActD, PD, CytD, Blebb and Y-27632 treated cultures (n=3 independent experiments; ***p<0.05 by Anova Tukey's multiple comparison test; error bars indicate SEM).

**Supplementary Figure 9. Key proteins for cytokinesis are activated during axokinesis.** Schematic representation of the molecular cascade activated during cytokinesis to form the contractil ring. Green shadowed boxes are proteins found in SLI after nerve damage and red shadowed boxes corresponds to drugs used in this study to inhibit the function of citokinesis-specific proteins during axonal fragmentation after nerve injury.
